# Anti-proliferative activity of the NPM1 interacting natural product avrainvillamide in acute myeloid leukemia

**DOI:** 10.1038/cddis.2016.392

**Published:** 2016-12-01

**Authors:** Vibeke Andresen, Bjarte S Erikstein, Herschel Mukherjee, André Sulen, Mihaela Popa, Steinar Sørnes, Håkon Reikvam, Kok-Ping Chan, Randi Hovland, Emmet McCormack, Øystein Bruserud, Andrew G Myers, Bjørn T Gjertsen

**Affiliations:** 1 Centre for Cancer Biomarkers (CCBIO), Department of Clinical Science, University of Bergen, Bergen, Norway; 2 Department of Chemistry and Chemical Biology, Harvard University, Cambridge, MA 02138, USA; 3 KinN Therapeutics, Bergen, Norway; 4 Department of Internal Medicine, Haukeland University Hospital, Bergen, Norway; 5 Department of Clinical Science, University of Bergen, Bergen, Norway; 6 Institute of Chemical and Engineering Sciences, Agency for Science, Technology, and Research (A*STAR), Singapore 138667, Singapore; 7 Centre of Medical Genetics and Molecular Medicine, Haukeland University Hospital, Bergen, Norway

## Abstract

Mutated nucleophosmin 1 (*NPM1*) acts as a proto-oncogene and is present in ~30% of patients with acute myeloid leukemia (AML). Here we examined the *in vitro* and *in vivo* anti-leukemic activity of the NPM1 and chromosome region maintenance 1 homolog (CRM1) interacting natural product avrainvillamide (AVA) and a fully syntetic AVA analog. The *NPM1*-mutated cell line OCI-AML3 and normal karyotype primary AML cells with *NPM1* mutations were significantly more sensitive towards AVA than cells expressing wild-type (wt) *NPM1*. Furthermore, the presence of wt p53 sensitized cells toward AVA. Cells exhibiting fms-like tyrosine kinase 3 (FLT3) internal tandem duplication mutations also displayed a trend toward increased sensitivity to AVA. AVA treatment induced nuclear retention of the NPM1 mutant protein (NPMc+) in OCI-AML3 cells and primary AML cells, caused proteasomal degradation of NPMc+ and the nuclear export factor CRM1 and downregulated wt FLT3 protein. In addition, both AVA and its analog induced differentiation of OCI-AML3 cells together with an increased phagocytotic activity and oxidative burst potential. Finally, the AVA analog displayed anti-proliferative activity against subcutaneous xenografted HCT-116 and OCI-AML3 cells in mice. Our results demonstrate that AVA displays enhanced potency against defined subsets of AML cells, suggesting that therapeutic intervention employing AVA or related compounds may be feasible.

Acute myeloid leukemia (AML) is a clinically and molecularly heterogeneous disease, characterized by the accumulation of malignant immature myeloid progenitor cells in bone marrow and peripheral blood.^1, 2, 3^ Nucleophosmin 1 (NPM1) is an essential multifunctional protein, including being a chaperone for pre-ribosomal particles, and regulation of the activity and stability of the tumor suppressor protein p53.^4, 5^ NPM1 is predominantly localized within the nucleoli and nucleoplasm of cells, but is a nucleocytoplasmic shuttle protein, where its nuclear export occurs through interaction with the nuclear export factor chromosome region maintenance homolog 1 (CRM1).^5, 6, 7^

*NPM1* mutations are observed in ~30% of all AML patients, and in ~50–60% of patients exhibiting a normal karyotype;^8, 9, 10, 11, 12^ such mutations associate with an improved prognosis, particularly among younger patients.^13, 14, 15, 16^ So far, most of the 50 *NPM1* mutations identified result in the displacement of this protein from the nucleus to the cytoplasm, consequently named NPMc+.^14, 15^ *NPM1* mutations are considered founder mutations in AML patients and highly stable during the course of disease.^16, 17, 18^ *NPM1*-mutated AML blasts are characterized by a unique microRNA signature, upregulated *HOX* genes and low or absent CD34 expression.^19, 20^

Another clinically significant mutation in AML is the internal tandem duplication (ITD) in the juxtamembrane domain of the fms-related tyrosine kinase 3 (FLT3) receptor; present in ~25% of AML patients (30% in normal karyotype AML), where ~40% of FLT3–ITD patients also comprising a *NPM1* mutation.^12, 21, 22^ The FLT3–ITD protein is constitutively active, resulting in increased cellular proliferation; this mutation is associated with resistance to chemotherapy, increased risk for disease relapse and overall decreased survival.^22^

Avrainvillamide (AVA) is a prenylated indole alkaloid initially isolated from *Aspergillus ochraceus*^23^ and has been the subject of intense synthetic endeavors.^24^ This anti-proliferative natural product was recently demonstrated to interact with NPM1wt, NPMc+ and CRM1.^25, 26^

In the present study, we investigated the effects of the natural product AVA and a fully synthetic AVA analog in leukemic cell lines, primary AML cells and in mouse xenograft subcutanous models. AVA displayed enhanced potency against cells expressing NPMc+, FLT3–ITD and wt p53, and indicated efficacy *in vivo*.

## Results

### AVA inhibits cellular proliferation and induces cell cycle arrest and apoptosis in AML cell lines

**Figure 1:**
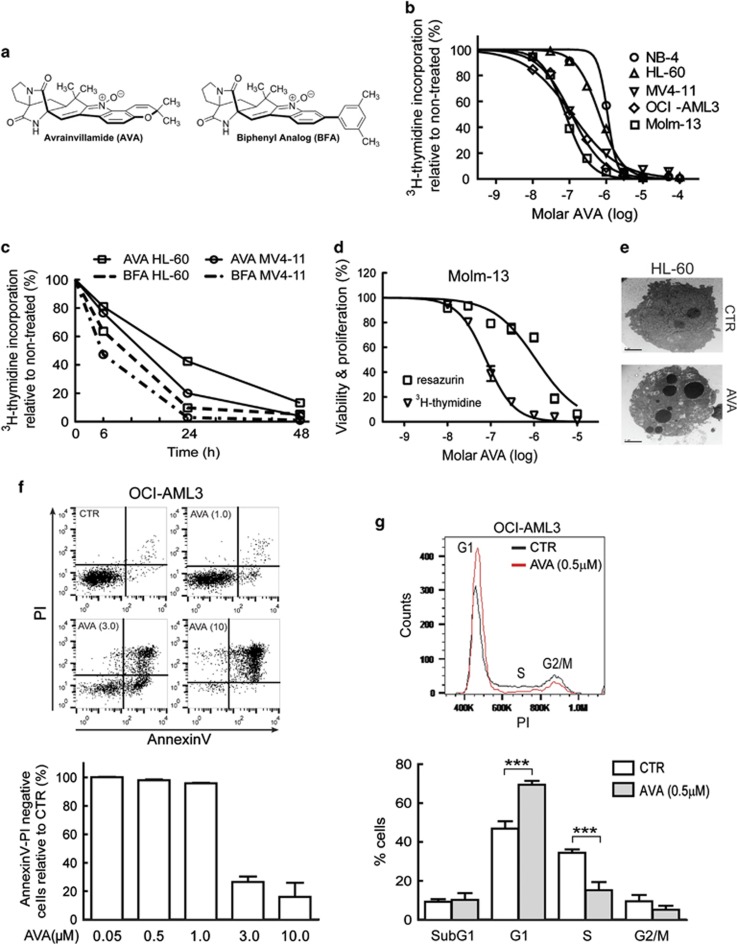
Effects of avrainvillamide on AML cell lines. (**a**) Structure of avrainvillamide (AVA) and AVA biphenyl analog (BFA). (**b**) NB4, HL-60, MV4-11, OCI-AML3 and Molm-13 cells were incubated with AVA (10 nM–30 *μ*M) for 24 h, (*n*=3–7), and proliferation was determined by ^3^H-thymidine incorporation. For all cell proliferation experiments, cells were incubated with ^3^H-thymidine for 6 h immediately before scintillation counting. Results represent the mean±S.E.M. of the values relative to vehicle controls. (**c**) HL-60 and MV4-11 cells were treated for 6, 24 or 48 h with 1 *μ*M AVA or its BFA (*n*=3–4), then proliferation was determined by ^3^H-thymidine incorporation. (**d**) Molm-13 cells were treated with varying concentrations of AVA (10 nM–30 *μ*M) for 24 h. The cells were either incubated with ^3^H-thymidine or resazurin for the final 4 h before scintillation counting or assessment of fluorescence emission, respectively. Results indicate the mean of the values relative to vehicle controls (*n*=3–4). Triangles and squares represent measured rates of proliferation and metabolic activity, respectively. (**e**) HL-60 cells were treated with DMSO (CTR) or 3 *μ*M AVA for 24 h and examined by transmission electron microscopy (scale bar, 2 *μ*m). (**f**) Histogram displayed for OCI-AML3 cells treated with DMSO (CTR) or 1.0, 3.0 and 10 *μ*M AVA for 24 h, stained with annexinV and propidium (PI) and analyzed by flow cytometry. AnnexinV/PI-negative cells are graphed as live cells normalized to CTR for all concentrations of AVA mean±S.D. (*n*=3). (**g**) Cell cycle analyses of OCI-AML3 cells treated for 24 h with 0.5 *μ*M AVA following fixation, PI staining and flow cytometry analysis. Representative histogram with G1, S and G2/M is shown and the subG1, G1, S and G2/M phases are graphed using mean±S.D. (*n* = 5, ***P<0.001). For all flow cytometric analyses least 10 000 events per sample were acquired

The anti-proliferative activity of AVA (Figure 1a) was studied in five different AML cell lines (Table 1). The IC_50_ values after 24 h treatment demonstrated that MV4-11, OCI-AML3 and Molm-13 cells were more sensitive compared with NB4 and HL-60 (Figure 1, Table 1). Proliferation kinetics were investigated by treating sensitive and less-sensitive cell lines with AVA or the biphenyl-modified AVA analog (BFA; Figure 1a) for 6, 24 and 48 h (Figure 1c). The IC_50_ value determined by the WST-1 assay in Molm-13 cells was 14.8-fold higher than IC_50_ measured by the ^3^H-thymidine-assay (Figure 1d); also found in HL-60, NB4 and MV4-11 (data not shown), suggesting that AVA affects the cellular proliferation before metabolic activity. The AML cell lines exhibited typical apoptotic morphology with condensed and fragmented nuclei, as determined by Hoechst33342 staining (data not shown) and transmission electron microscopy (Figure 1e). OCI-AML3 cells treated with increasing concentrations of AVA for 24 h demonstrated a rapid decrease of annexin/PI-negative cells (live cells) at 3 *μ*M compared with 1* μ*M (Figure 1f). OCI-AML3 cells treated with 0.5 *μ*M AVA for 24 h resulted in cell cycle G1-phase arrest (Figure 1g), also found in MV4-11 cells (Supplementary Figure 1).

### AVA displays anti-proliferative activity that associates with NPM1 and FLT3 mutational status in AML patient cells

**Figure 2:**
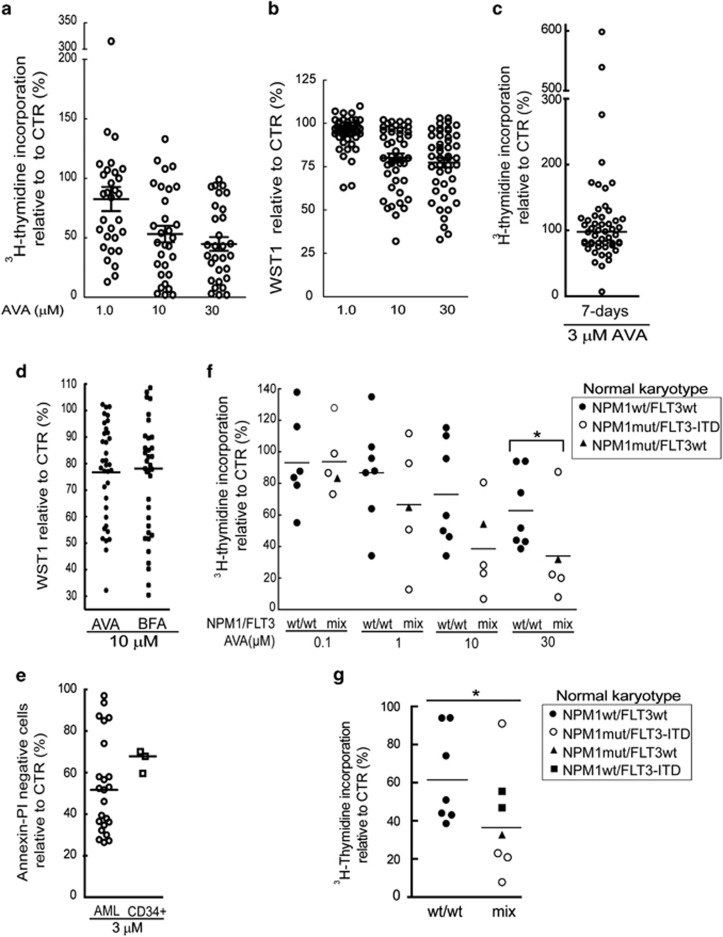
Effects of avrainvillamide on primary AML patient cells. Primary AML cells were incubated with avrainvillamide (AVA; 10 nM–300 *μ*M) for 24 h and proliferation assayed by (**a**) ^3^H-thymidine incorporation or (**b**) metabolic activity determined by the WST-1 assay. Results are shown for 1.0, 10 and 30 *μ*M AVA treatment. (**c**) Primary AML cells were incubated with a cytokine cocktail (FLT3 ligand, SCF and GM-CSF) containing DMSO or AVA (3 *μ*M) for 7 days. Proliferation was assayed by ^3^H-thymidine incorporation. Circles represent the mean values relative to vehicle controls; horizontal bar indicates the median of all 52 values. (**d**) Primary cells from 35 AML patients were treated with AVA or BFA (10 *μ*M) for 24 h. Dots or squares represent the mean values relative to vehicle controls compared with untreated controls. Horizontal bars represent the median values. (**e**) Primary cells from 23 AML patients and three umbilical cord CD34+ blood samples were treated with AVA (3 *μ*M) for 24 h. Cells were stained with annexinV/PI and analyzed by flow cytometry. Circles represent the mean values relative to vehicle controls. Horizontal bars indicate the median values. (**f**) Primary AML cells with normal karyotype and either wild-type NPM1 (wt, *n*=7) or mutated NPM1 (mut, *n*=5) were treated with AVA (0.1, 1.0, 10 and 30 *μ*M) for 24 h and assayed by ^3^H-thymidine incorporation. Symbols represent the mean values relative to vehicle controls. Wt NPM1, wt FLT3 (●), mutant NPM1, FLT3–ITD (○) and mutant NPM1, wt FLT3(▴). Horizontal bars represent the median values (*P<0.05). (**g**) The same patient samples from **f**, including two additional FLT3–ITD, wt NPM1 AML patient samples (▪) were incubated with 30 *μ*M AVA and assayed by ^3^H-thymidine incorporation. Wt NPM1, wt FLT3 (▴), mutant NPM1, FLT3–ITD (○) and mutant NPM1, wt FLT3(▴). Horizontal bars represent the median values (*P<0.05)

AML patient samples (Supplementary Table 1) were treated for 24 h with 10 nM–100 *μ*M AVA (Figure 2a; 1, 10 and 30 *μ*M presented). We observed a highly variable anti-proliferative response with a median IC_50_ value of 7.1 μM (Supplementary Table 2; Supplementary Figure 2a), similarly using the WST-1 assay (Figure 2b). Primary AML patient samples (Supplementary Table 3) incubated for 7 days with FLT3 ligand, GM-CSF and SCF also responded highly variable (Figure 2c). Some of the primary AML cells showed increased proliferation at lower doses a phenomena also described for other anti-cancer drugs.^27, 28^ No significant differences in the mean following AVA or BFA treatment analog in 35 AML patient samples were found by the WST-1 assay (Figure 2d), or by the ^3^H-thymidine-assay (data not shown). We then investigated if AVA displayed enhanced potency toward malignant cells compared with healthy umbilical cord blood CD34+ cells from three donors (Figure 2e) and healthy PBMCs from five donors (Supplementary Figures 2b,c). If the CD34+ cells represented a homogeneous AVA response, one sensitive and one less-sensitive group of AML cells were observed. However, most likely attributed to the heterogeneous response by the primary AML cells, no significant differences were observed (Figure 2e). Healthy PBMCs demonstrated a homogenous response and appeared more sensitive compared with the CD34+ cells with an IC_50_ value of <3 *μ*M (Supplementary Figures 2b, c).

To identify sensitive AML patient-derived cells, we sought to correlate the effects of AVA with biological characteristics. AVA treatment inhibited AML-M5 to a greater extent than AML-M1 (Supplementary Figure 3a). Similar results were obtained in the 7-days assay using 1 *μ*M AVA (*P*=0.041, data not shown). We also observed an association between AVA sensitivity and expression levels of CD15, CD14 and CD11c (Supplementary Figures 3b–d). As AML-M5 cells exhibit a more mature phenotype and presumably higher basal proliferation activity,^29^ we investigated proliferation and AVA sensitivity. Indeed, we found a significant association between proliferation rates and proliferation inhibition in AML patient cells (Supplementary Figure 3e). We did not, however, find significant differences in basal proliferation rates between AML-M1 and AML-M5 patient cells or cells with high or low CD15 expression (data not shown), indicating that factors besides proliferation rate play a sensitizing role toward AVA.

Mutations in the *NPM1* and *FLT3* genes constitute the largest groups of genetic changes in AML patients with normal karyotype.^12^ *NPM1* mutations are primarily associated with AML-M4 and AML-M5^19, 30^ and almost always result in a change of reading frame in the C-terminal domain of the NPM1 mutant and a cytoplasmic localization in AML cells.^14^ As the NPM1-mutated OCI-AML3^31^ cell line demonstrated enhanced sensitivity towards AVA that interacts with the C-terminal domain of both wild-type (wt) and mutant NPM1,^25, 26^ and the BFA with the C-terminal of NPM1 (personal communication; https://dash.harvard.edu/handle/1/17467289); we investigated if NPM1 mutational status was associated with AVA sensitivity. We studied the anti-proliferative effects of AVA in 12 normal karyotype AML patient samples. Cells bearing *NPM1* mutations were significantly more sensitive toward AVA than cells expressing *NPM1*wt only (Figure 2f). As four of the NPMc+ patient samples also contained an *FLT3–ITD* mutation, we sought to determine if *FLT3* mutational status similarly affected AVA sensitivity. Significance were still observed after including two additional normal karyotype AML patient samples with *FLT3–ITD* and *NPM1*wt and cells with FLT3–ITD mutations showed a distinct trend toward significance (Figures 2g; *P*=0.073) suggesting an AVA-sensitizing role for *FLT3–ITD* mutations.

### p53 sensitizes AML cells toward AVA treatment

**Figure 3:**
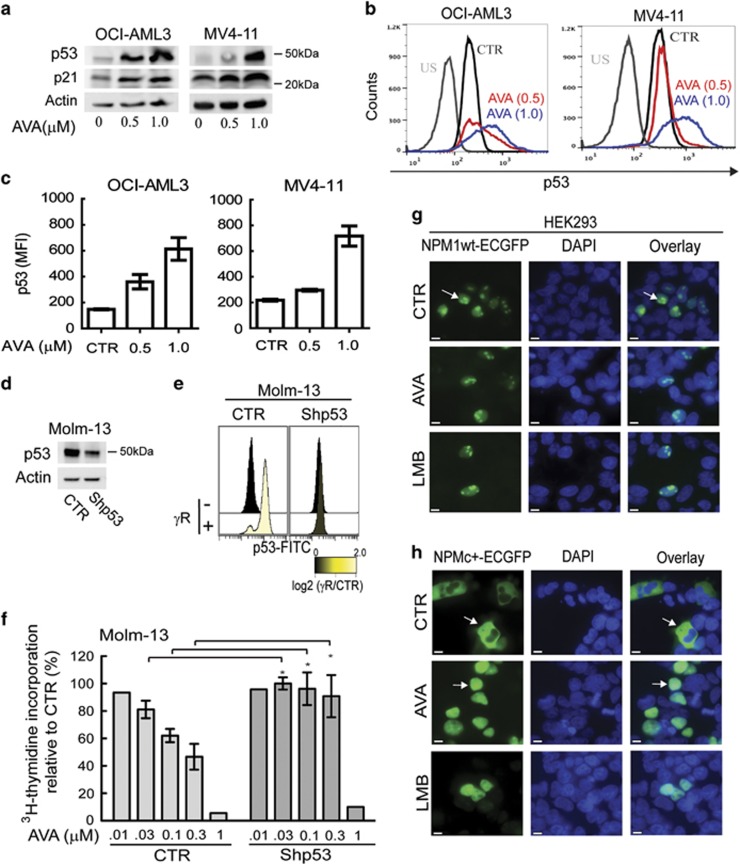
The presence of wild-type p53 sensitizes cells towards avrainvillamide treatment. (**a**) Immunoblots showing p53, p21 and actin (loading control) expression levels in OCI-AML3 and MV4-11 cells treated with avrainvillamide (AVA; 0, 0.5 and 1.0 *μ*M) for 24 h. (**b**) Flow cytometry analysis of p53 expression in OCI-AML3 and MV4-11 cells treated with DMSO (CTR) or 0.5 *μ*M AVA for 24 h. Representative results from a single experiment are shown. US, unstained. (**c**) Flow cytometric median fluorescence intensity (MFI) quantification of p53 (unstained subtracted, *n*=3) in OCI-AML3 and MV4-11 cells. Results represent mean±S.E.M. (**d**) Molm-13 cells were transduced with empty vector (CTR) or shp53 then immunoblotted for p53 expression. Actin is shown as a loading control. (**e**) Flow cytometry analyses of p53 expression levels in non-irradiated and gamma-irradiated Molm-13 cells transduced with empty vector (CTR) or shp53. (**f**) Proliferation of Molm-13 cells following transduction with empty vector (CTR) or shp53 and treatment with AVA (0.01 (.01), 0.03 (.03), 0.1, 0.3 or 1 *μ*M) for 24 h. Results represent mean±S.E.M. of the values relative to vehicle controls (*n*=4; *P<0.05). HEK293 cells were transfected with (**g**) NPM1wt-ECGFP or (**h**) NPMc+-ECGFP, and treated with DMSO (CTR) or AVA (0.5 *μ*M) for 24 h or LMB (10 nM) for 3 h. Cells were fixed and analyzed by fluorescence microscopy. All transfection experiments were conducted at least three times; representative images are shown. Arrows indicate localization of NPM1wt and NPMc+ (DAPI indicates nuclei; scale bar, 10 *μ*m)

As NPM1 influences the activity and stability of the p53 protein^5^ and given the association between *TP53* mutations, p53 protein expression level, isoform profile and long-term survival in AML patients,^32, 33, 34^ we examined the connection between AVA and p53. AVA induces p53 expression in certain cancer cell lines;^26^ furthermore, wt p53 AML cell lines were more sensitive compared with p53 null or p53 mutated (Figure 1b; Table 1). Subsequent immunoblot and flow cytometry experiments revealed that AVA treatment increased p53 and p21 protein levels in OCI-AML3 and MV4-11 cells (Figures 3a–c). To investigate the potential roles of p53 in AVA-induced anti-proliferation, Molm-13 cells transduced with either short hairpin RNA against p53 (shp53) or empty control vector (CTR) were studied.^35^ Cells with shp53 showed ~70% reduced p53 expression and p53 activation after gamma-irradiation compared with CTR cells (Figures 3d and e). The shp53 cells were significantly less sensitive toward AVA compared with CTR cells (Figure 3f), demonstrating an AVA-sensitizing role of p53.

We then investigated whether AVA influenced the subcellular localization and expression levels of wt NPM1 and NPMc+ proteins. As a positive control, we included the CRM1 inhibitor, leptomycin B (LMB), causing nuclear retention of NPMc+ proteins.^36^ HEK293 cells transfected with NPM1wt-ECGFP or NPMc+-ECGFP plasmids were treated with DMSO, AVA or LMB. The NPM1wt-ECGFP protein localized predominantly within the nuclei and nucleolus of control cells and no change was observed in treated cells (Figure 3g). On the other hand, the NPMc^+^-ECGFP protein localized to the cytoplasm of control cells, but relocated to the nuclei after AVA or LMB treatment (Figure 3h).

### AVA causes nuclear retention and downregulation of NPMc+ accompanied by decreased expression of CRM1 and FLT3 in AML cells

**Figure 4:**
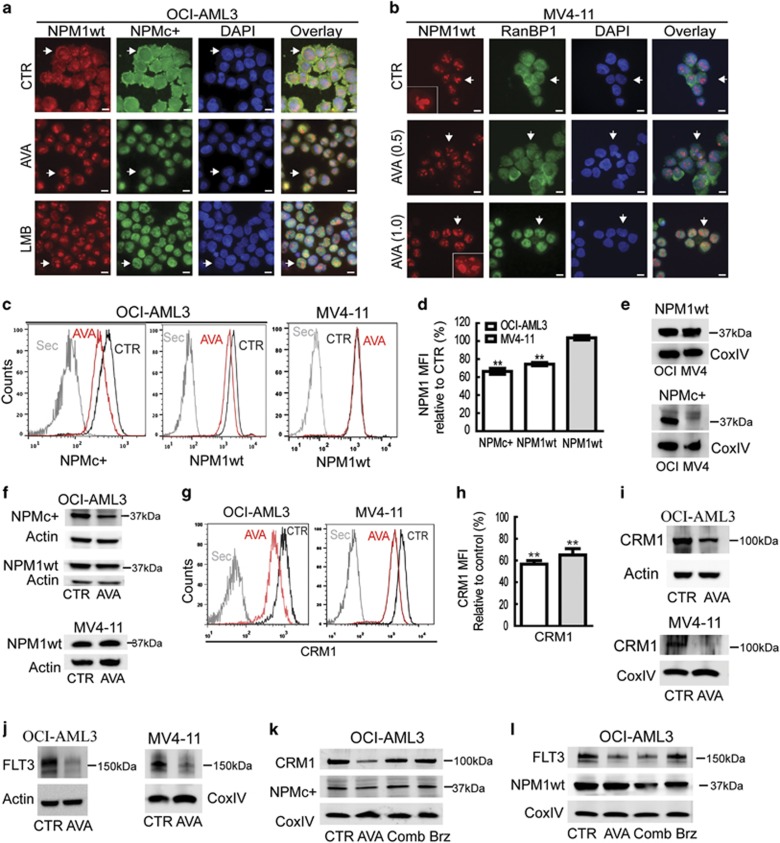
Avrainvillamide treatment causes nuclear retention of NPMc+ proteins and reduced expression levels of NPMc+, CRM1 and FLT3 proteins in AML cell lines. (**a**) OCI-AML3 cells were treated DMSO (CTR) or avrainvillamide (AVA; 0.5 *μ*M) for 24 h or LMB (10 nM) for 3 h, then cytospun onto coverslips, fixed, and stained with NPM1wt- and NPMc+-specific antibodies. (**b**) MV4-11 cells were treated with DMSO (CTR) or AVA (0.5 *μ*M and 1.0 *μ*M), cytospun onto coverslips, fixed, and stained with NPM1wt and RanBP1 antibodies. All immunofluorescence experiments were conducted at least three times; representative images are shown and arrows indicate described localization and zoomed cells for MV4-11. (DAPI indicates nuclei; scale bar, 10 *μ*m). (**c**) OCI-AML3 and MV4-11 cells were treated with AVA (0.5 *μ*M) for 24 h and analyzed by flow cytometry using specific antibodies against NPMc+ and NPM1wt as indicated. Results from representative experiments are shown. (Sec=secondary antibody only). (**d**) Flow cytometry median fluorescence intensity (MFI) quantification of NPM1 expression levels in OCI-AML3 and MV4-11 cells. Results represent the median±S.E.M. of three independent experiments (**P<0.01). (**e**) Validation of NPM1 antibody specificity by immunoblotting using cell lysates of OCI-AML3 (OCI) and MV4-11 (MV4) incubated with NPM1wt and NPMc+ antibodies. (**f**) Immunoblots for NPMc+ expression in OCI-AML3 cells and NPM1wt expression in OCI-AML3 and MV4-11 cells incubated with AVA (0.5 *μ*M) for 24 h. (**g**) CRM1 expression as determined by flow cytometry in OCI-AML3 and MV4-11 cells after incubation with AVA (0.5 *μ*M) for 24 h, representative image is shown and (**h**) flow cytometry MFI quantification of the median±S.E.M. of three independent experiments relative to control (*n*=3, **P<0.01; Sec=secondary antibody only). (**i**) CRM1 expression after incubation with AVA (0.5 *μ*M; OCI-AML3, 1.0 μM; MV4-11) for 24 h, as determined by immunoblotting. (**j**) FLT3 expression after incubation with AVA (0.5 *μ*M; OCI-AML3, 1.0 *μ*M; MV4-11) for 24 h, as determined by immunoblotting. (**k** and **l**) OCI-AML3 cells were treated with either DMSO (CTR), AVA alone (0.5 *μ*M, 24 h), AVA (0.5 *μ*M, 24 h) with bortezomib (Comb; 50 nM, final 8 h) or bortezomib (Brz; 50 nM, final 8 h) alone and then immunoblotted for NPMc+, CRM1, FLT3 and NPM1wt. Actin and CoxIV were used as loading controls for the immunoblots

We next examined the effect of AVA on endogenous expression of NPMc+ in OCI-AML3 cells by immunostaining using NPM1wt- and NPMc+-specific antibodies. NPM1wt concentrated in the nucleoli and nucleoplasm of control cells, but was also observed in the cytoplasm, presumably due to hetero-oligomer formation,^37^ whereas NPMc+ predominantly localized to the cytoplasm (Figure 4a). Following AVA and LMB treatment, NPM1wt exclusively localized to the nucleoli and nucleoplasm, whereas NPMc+ re-localized from the cytoplasm to the nucleus (Figure 4a). The effect of AVA on cells expressing NPM1wt only was assessed in MV4-11 cells. RanBP1, another nucleocytoplasmic shuttle protein and CRM1 interacting protein, was used as a potential positive control for the nuclear export inhibiting the effect of AVA. The localization of NPM1wt and RanBP1 was influenced by 1 *μ*M AVA treatment, resulting in nuclear localization of RanBP1 and in addition to nucleolar, NPM1wt was found in nucleoplasmic dots (Figure 4b), indicating a connection between cell cycle arrest, p53 and nucleolar stress, as reported previously.^38^

Quantification of NPM1wt and NPMc+ protein levels following AVA treatment, using flow cytometry, revealed a significant reduction of NPMc+ in OCI-AML3 cells (Figures 4c and d). AVA treatment also reduced NPM1wt levels in OCI-AML3 cells; however, a similar decrease was not observed in MV4-11 cells (Figures 4c and d). Specificity of the anti-NPM1 antibodies was demonstrated by immunoblotting (Figure 4e), before the flow cytometry results were confirmed, except the reduction of NPM1wt in OCI-AML3 cells that appeared less pronounced by immunoblotting (Figure 4f).

As the nuclear export of NPMc+ and NPM1wt proteins is mediated by CRM1 and AVA interacts with NPM1 and CRM1,^25^ we investigated the effects of AVA on CRM1 activity and expression levels. Following 0.5 *μ*M AVA RanBP1 was retained in the nucleus of OCI-AML3 cells (data not shown), in agreement with the previous results in the MV4-11 cells and in the non-leukemic cells HCT-116.^25^ Furthermore, we observed CRM1 downregulation in OCI-AML3 and MV4-11 cells by flow cytometry and this was confirmed by immunoblotting, most prominent at 1 *μ*M concentration for MV4-11 cells (Figures 4g–i).

Recently, FLT3 downregulation has been reported upon treatment with a CRM1 inhibitor^39^ and we therefore assessed the effects of AVA on FLT3 expression and localization in OCI-AML3 cells and MV4-11 cells. AVA reduced FLT3 levels in OCI-AML3 and FLT3–ITD expression in MV4-11 cells (at 1 *μ*M) as determined by immunoblotting (Figure 4j). No change in subcellular localization was found for FLT3wt protein in OCI-AML3 cells, following AVA treatment (data not shown). When OCI-AML3 cells were co-treated with AVA and the proteasome inhibitor bortezomib, the AVA-induced CRM1 and NPMc+ degradation was reversed, but not the FLT3 degradation (Figures 4k and l).

**Figure 5:**
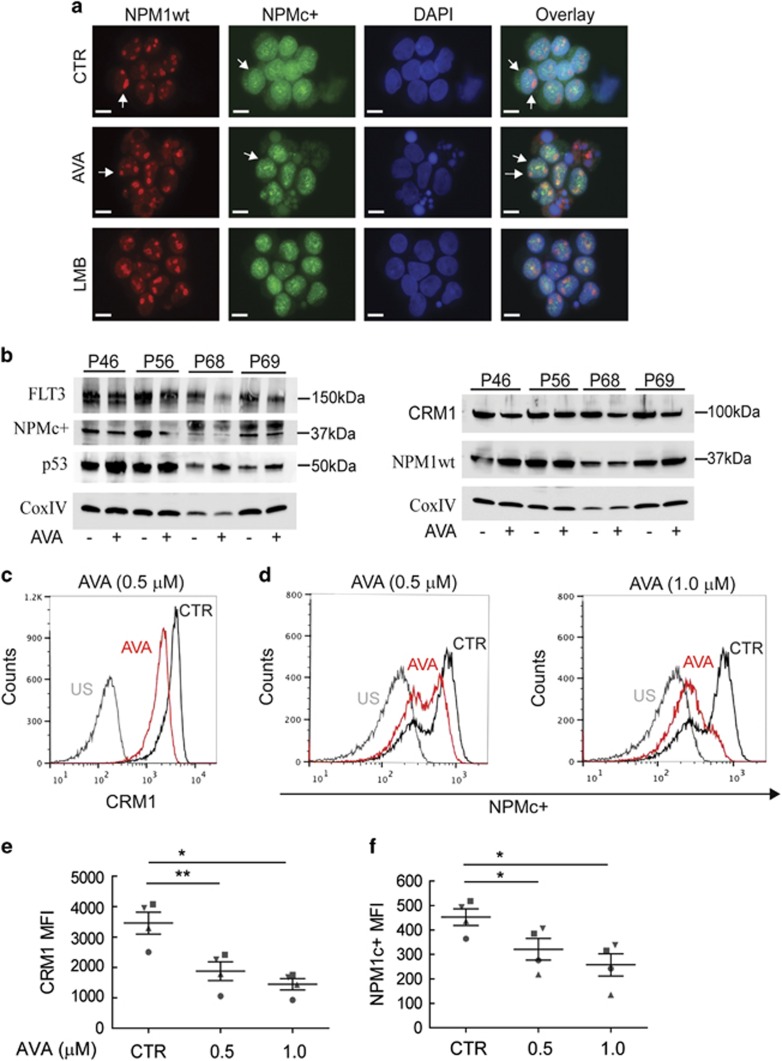
Avrainvillamide treatment decreases expression levels of NPMc+, CRM1 and FLT3 proteins in AML primary patient cells. (**a**) Four primary AML samples (Supplementary Table 5) were treated with DMSO (CTR), avrainvillamide (AVA; 0.5 *μ*M and 1.0 *μ*M) for 24 h or LMB (10 nM) for 3 h. Cells were then cytospun onto coverslips, fixed and stained using NPMc+- and NPM1wt-specific antibodies. Results from one representative patient sample (P46) are shown (DAPI indicates nuclei; scale bar, 10 *μ*m). (**b**) AML patient samples (Supplementary Table 4) were treated with DMSO (CTR), AVA (0.5 *μ*M) for 24 h or LMB (10 nM) for 3 h, lysed and immunoblotted for CRM1, NPMc+, p53, FLT3, NPM1wt and CoxIV expression (loading control). (**c**–**f**) AML patient cells (Supplementary Table 5) were treated with DMSO (CTR) or AVA (0.5 *μ*M and 1.0 *μ*M) for 24 h, fixed and analyzed by multiplexed flow cytometry for CRM1 (**c**), and NPMc+ (**d**) expression by gating on living cells (stained with an aminoreactive dye before permeabilization), specifically analyzing AML blasts, as determined by the use of anti-CD45- (negative) and anti-CD33- (positive) specific antibodies. Results from one representative patient sample are shown. US, unstained. (**e**) Flow cytometric median fluorescence intensity (MFI) quantification of CRM1 and (**f**) NPMc+ expression in the four primary AML patient samples, results represent mean±S.E.M. Comparisons between treated and control samples were conducted using a paired Student's *t*-test (*P<0.05, **P<0.01)

We further studied the effects of AVA on four primary AML samples with normal karyotype, wt FLT3 and mutated NPM1 (Supplementary Table 4). The AML patient samples were treated with DMSO, AVA or LMB then analyzed by immunofluorescence, immunoblotting and flow cytometry (Figure 5). In contrast to our observations in the OCI-AML3 cell line, NPMc+ did not exclusively accumulate within the cytoplasm of patient-derived AML blasts. Rather, the NPMc+ protein localized to the cytoplasm, diffusely throughout the nucleoplasm and in nucleoplasmic speckles. The NPMc+ protein was apparently excluded from nucleoli, contrasting the predominant nucleolar localization of the NPM1wt protein (Figure 5a). Both AVA and LMB treatment resulted in the reduced cytoplasmic NPMc+, and a nucleoplasmic localization was observed, whereas no change in subcellular localization was found for NPM1wt with any treatment (Figure 5a). Immunoblotting revealed that all four AVA-treated patient samples showed reductions in NPMc+, FLT3 and CRM1 protein levels, increased protein levels of p53 and no apparent change in NPM1wt levels (Figure 5b). Flow cytometry confirmed reduced levels of NPMc+ and CRM1 (Figures 5c–f) and p53 increase (data not shown). Taken together our data demonstrate that AVA influences the subcellular localization of NPM1wt and NPMc+ proteins, and modulates protein levels of NPMc+, CRM1, FLT3 and p53 in AML cell lines and primary AML cells.

### AVA and the AVA analog induce cellular differentiation, increase phagocytosis and ROS capacity

**Figure 6:**
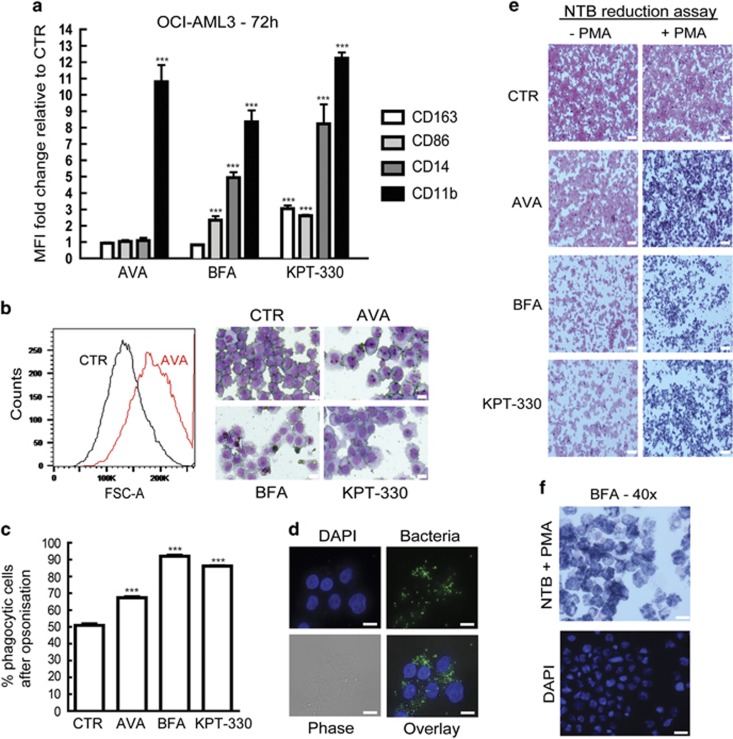
Avrainvillamide and BFA induce differentiation, increase phagocytosis and oxidative burst of OCI-AML3 cells. (**a**) OCI-AML3 cells were treated for 72 h with DMSO (CTR), AVA (0.5 *μ*M), BFA (0.5 μM) or KPT-330 (0.1 *μ*M) before live cells were stained and analyzed by flow cytometry for CD163, CD86, CD14 and CD11b expression by gating on living cells. Fold changes (mean±S.D.) relative to controls are shown (*n*=3). Comparisons between treated and control samples were conducted using an unpaired Student's *t*-test (***P<0.001). (**b**) OCI-AML3 cells were treated for 72 h with DMSO (CTR), avrainvillamide (AVA; 0.5 *μ*M), BFA (0.5 *μ*M) or KPT-330 (0.1 *μ*M) and analyzed by flow cytometry and cytospun and stained with May-Grunwald-Giemsa before analyzed by microscopy (Zeiss axio Observer A1) using a × 40 objective lens. A representative histogram for AVA-treated cells is shown to the left and representative May-Grunwald-Giemsa stained images are shown (*n*=3). Scale bars, 10 μm. (**c**) OCI-AML3 cells treated with DMSO (CTR), AVA (0.5 *μ*M), BFA or KPT-330 for 72 h followed by phagocytosis assay with opsonization of fluorescent bacteria. Quantification of increase in phagocytotic cells compared with CTR (*n*=3), results represent mean±S.D. (***P<0.001). (**d**) Localization of fluorescently labeled bacteria in 72 h BFA-treated OCI-AML3 cells stained with DAPI. Images were captured using Zeiss Axio ObserverZ1, × 63 oil objective. Scale bars, 10 μm. (**e**) OCI-AML3 cells were treated with DMSO (CTR), AVA (0.5 *μ*M), BFA (0.5 *μ*M) or KPT-330 (0.1 *μ*M) for 72 h before 2 × 10^5^ cells were incubated with nitroblue tetrazolium (NTB—reaction with ROS produces a dark blue color) and stimulated with PMA for 30 min at 37 °C. Cells were cytospun and counterstained with Safranin O and images were captured using Zeiss Axio Observer A1, × 10 objective (*n*=3). Scale bars, 50 μm. (**f**) BFA-treated cells from (e) were stained with additional DAPI. Images were captured using Zeiss Axio Observer A1, × 40 objective. Scale bars, 20 *μ*m

As compounds interacting with CRM1 or NPM1 induce cellular differentiation,^39, 40^ we sought to determine if AVA caused differentiation. OCI-AML3 cells were treated for 72 h with AVA, BFA or the CRM1 inhibitor KPT-330, then subjected to flow cytometric analysis for expression levels of common myeloid differentiation markers (CD11b, CD14, CD86, CD163, HLA-DR, CD16 and CD40). All three compounds significantly induced CD11b expression relative to controls; furthermore, BFA increased CD86 and CD14 expression, whereas KPT-330 significantly induced CD163, CD86 and CD14 (Figure 6a; Supplementary Figure 4). BFA induced an increase in CD86 and CD14 expression, not observed for AVA alone, which could indicate a functional difference. No changes in expression were observed for HLA-DR, CD16 or CD40 under any treatment conditions.

In addition, flow cytometric forward-side scatter analysis revealed an increase in cellular size upon exposure to all three compounds; this was verified by May-Grünwald-Giemsa staining demonstrating larger cells with increased cytoplasm (Figure 6b). As CD11b expression was increased, we next assessed the phagocytic potential of these cells without and with human serum opsonisation in a fluorescent phagocytosis assay. All treatments resulted in increased number of phagocytic cells after opsonisation as compared with the control cells (which themselves also demonstrated phagocytic activity), indicating IgG- or complement-mediated binding of bacteria to the cells (Figure 6c). Fluorescence microscopy indicated increased number of bacteria associated with treated cells (Figure 6d). Addition of trypan blue to quench fixed extracellular bacteria, but not the internalized, revealed that the treated, differentiated cells contained increased degree of internalized bacteria relative to undifferentiated control cells (data not shown). We next investigated the oxidative burst activity by adding a reactive oxygen species (ROS) probe to this system. Treated cells exhibited higher oxidative capacity compared with control cells; however, these results were found to be insensitive to opsonisation of the Gram-positive bacteria. Notably, the same result was found even without bacteria, indicating that the differentiated cells exhibited higher basal oxidative capacity (data not shown). To further investigate this, OCI-AML3 cells were treated for 72 h and evaluated using a nitroblue tetrazolium (NBT) reduction assay where ROS reaction with NBT results in a dark blue precipitate. Following phorbol 12-myristate 13-acetate (PMA) stimulation, treated and differentiated cells more effectively generated ROS compared with control cells, providing evidence for increased oxidative burst capacity of treated cells (Figures 6e and f).

### AVA analog treatment reduces tumor growth *in vivo*

**Figure 7:**
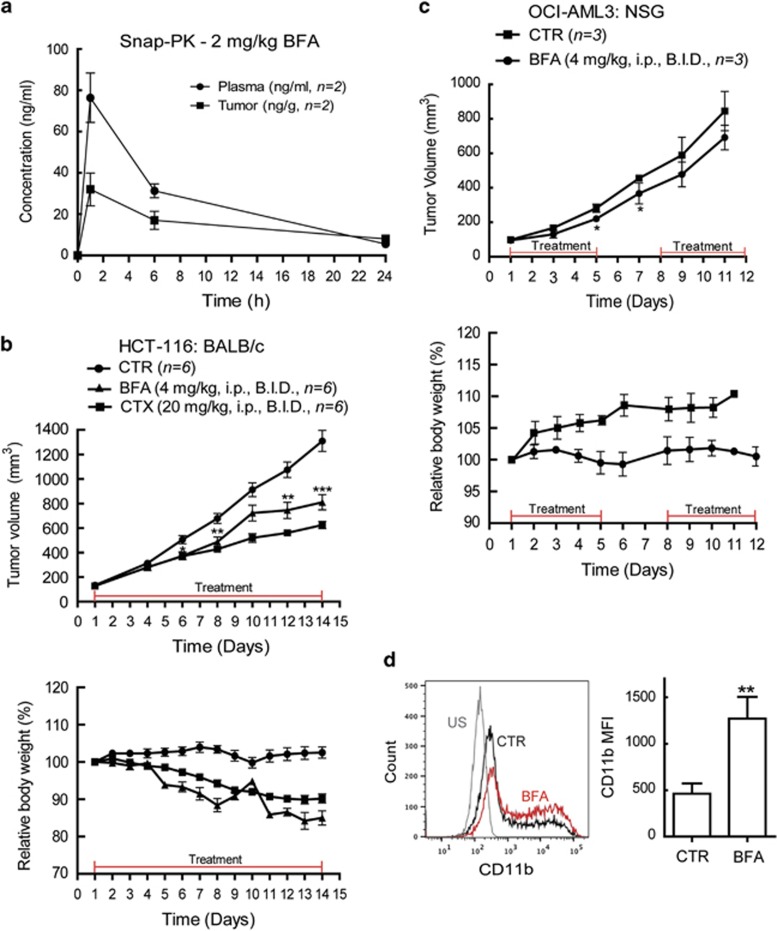
BFA treatment reduces the tumor growth in xenograft mouse models. (**a**) Snap-PK for 2 mg/kg BFA concentration (ng/ml) in plasma and tumor of BALB/c nude mice implanted subcutaneously with HCT-116 cells (*n*=2) measured after 1, 6 and 24 h. (**b**) BALB/c nude mice xenografted subcutaneously with HCT-116 cells were treated BID i.p., when tumor sizes reached ~100 mm^3^ with vehicle control (*n*=6), BFA 4 mg/kg (*n*=6) or CTX 20 mg/kg (*n*=6) as indicated for 14 days. The tumor sizes were measured and relative weight change (%) calculated. Comparisons (mean±S.E.M.) between tumors from treated and control mice were conducted using an unpaired Student's *t*-test (*P<0.05, **P<0.01). (**c**) NSG mice xenografted subcutaneously with OCI-AML3 cells were treated BID i.p., when tumor sizes reached ~100 mm^3^ with vehicle control (*n*=3) or BFA 4 mg/kg (*n*=3), as indicated and the tumor sizes were measured and relative weight change (%) calculated. Comparisons (mean±S.E.M.) between tumors from treated and control mice were conducted using an unpaired Student's *t*-test (*P<0.05). (**d**) Live tumor-derived cells were stained and analyzed by flow cytometry for CD11b expression (median fluorescence intensity (MFI)) by gating on living cells (PI or TO-PROIII negative). Representative histogram is shown at the left. Fold changes (mean±S.D.) relative to controls (CTR) are shown (*n*=3 tumors). Comparisons between control and treated tumor-derived cells were conducted using an unpaired Student's *t*-test (**P<0.01). US, unstained

We first assessed the *in vivo* activity of 2 mg/kg BFA in BALB/c nude mice. Snapshot pharmacokinetics (PKs) revealed an increase in the accumulation of BFA in plasma and in subcutaneous HCT-116 xenografted tumors 1 h after administration intraperitoneal (i.p.) injection. After 6 h, BFA plasma and tumor concentrations decreased to ~50%, indicating a need for a twice a daily (BID) administration (Figure 7a). An initial toxicity screen, based on the lack of significant weight loss, revealed that 4 mg/kg BFA (BID, i.p.) was well tolerated; however, two of six animals died when dosing was increased to 10 mg/kg BID (data not shown). For tumor growth inhibition experiments, BALB/c nude mice were subcutaneously implanted with HCT-116 cells; as we previously investigated AVA effect on this cell line.^25^ When tumors reached ~100 mm^3^, treatment was initiated for 14 days with vehicle control, BFA (4 mg/kg BID, i.p.) or cyclophosphamide (CTX, 20 mg/kg BID, i.p.). Both BFA and CTX treatment resulted in a significant tumor growth inhibition (Figure 7b), although also exhibiting low toxicity as measured by changes of relative body weight. CTX inhibited the tumor growth to a greater extent than treatment with BFA (Figure 7b).

Finally, we investigated the efficacy of BFA against a xenograft OCI-AML3 tumor model in NOD/SCID IL2r*γ*^null^ (NSG) mice.^41^ An initial toxicity screen revealed that 4 mg/kg BFA (BID, i.p.) was well tolerated, whereas higher concentrations were toxic (data not shown). Thus, NSG mice were injected subcutaneously with OCI-AML3 cells and treatment was initiated when tumors reached ~100 mm^3^. Vehicle control or BFA (4 mg/kg, BID, i.p.) were administered on a 5-days on, 2-days off schedule for 2 weeks. BFA-treated animals showed slightly reduced tumor growth rates as compared with vehicle-treated animals and no significant weight loss was observed during the treatment period (Figure 7c). Subcutaneous tumors were collected at euthanization and cells were cryopreserved. Tumor cells of control (*n*=3) and treated (*n*=3) mice were investigated by flow cytometric analysis for expression levels of common myeloid differentiation markers and CD11b expression was significantly increased in the tumor cells from the BFA-treated mice (Figure 7d). However, no increased ROS capacity was found in any of the tumor-derived cells following the NBT reduction assay (data not shown). Also, no change was found in cellular morphology after May-Grünwald-Giemsa staining, NPMc+ localization after immunostaining, or CRM1 or FLT3 protein expression by immunoblotting (data not shown).

## Discussion

AML cells with NPM1 mutations exhibited increased sensitivity toward AVA treatment, a trend also observed in AML cells with FLT3–ITD mutations. The last observation is of particular interest, as 40% of AML patients exhibiting *NPM1* mutations concurrently contain *FLT3–ITD* mutations and this is associated with early relapse and poorer prognosis compared with AML patients with *NPM1* mutations only.^12, 21^

AVA-induced G_1_-phase cell cycle arrest in AML cells consistent with an increase in p53 and p21 levels demonstrated previously to directly or indirectly induce cell cycle arrest.^42^ Although the presence of wt p53-sensitized AML cells toward AVA, higher doses inhibited proliferation of p53-reduced, p53-null or p53-mutated cells. *TP53* mutations have been reported mutually exclusive with *NPM1* mutation and *FLT3–ITD,*^43, 44, 45^ underscoring the probability for AVA sensitivity in AML patients with NPM1 and FLT3–ITD.

We additionally found that AVA treatment resulted in proteasome-mediated degradation of NPMc+, CRM1 and a reduction of FLT3 through an unknown mechanism. Interestingly, siRNA-mediated reduction of wt NPM1 in OCI-AML3 cells has previously been reported to reduce FLT3 and NPMc^+^ levels, and cause increased cellular sensitivity toward retinoic acid and cytarabine, suggesting a connection between NPM1 and FLT3 regulation.^40^

AVA did not affect the subcellular localization of the NPM1wt-ECGFP protein in transfected HEK293 cells; however, the NPMc+-ECGFP protein was retained in the nuclei. Similarly, AVA treatment did not affect the localization of wt NPM1 in OCI-AML3 cells or primary AML blasts, but induced nuclear retention of the cytoplasmic NPMc+ protein. AVA also induced nuclear retention of other CRM1-dependent cargo proteins, such as RanBP1, supporting a direct interaction between AVA and CRM1.^25^

The selective CRM1 inhibitor LMB prevents the nuclear export of CRM1 cargo proteins by covalent modification of CRM1 Cys^528^.^46^ However, LMB and other CRM1 inhibitors have been demonstrated too toxic for use *in vivo*.^46^ Recently, novel orally available, selective inhibitors of nuclear export have been developed,^39, 47^ which also interact with Cys^528^ of CRM1, inhibit nuclear export of CRM1 cargo proteins and exhibit anti-cancer activity *in vitro* and *in vivo* models of leukemia and lymphoma.^39, 48, 49, 50, 51^ Interestingly, AML cell lines and primary AML cells treated with the CRM1 inhibitor KPT-185 shared several phenotypic similarities with AVA-treated cells, including induction of p53 and downregulation of both FLT3 and CRM1.^39^ In addition, we found that AVA and the AVA analog (as well as KPT-330) induced morphological and immunophenotypic differentiation of OCI-AML3 cells. The effects of AVA on cellular differentiation are likely directly linked to the observed increase in p53 expression, as reported for KPT-185.^39^ We further demonstrated the cellular functionality associated with the differentiated cells, such as increased oxidative burst capacity and phagocytosis. This clearly unveils the important therapeutic potential hidden in the discovery of novel compounds with the capacity to induce differentiation in AML cells.^52^

Finally, we found a modest anti-proliferative effect of the AVA analog *in vivo*. However, calculation of AVA analog concentration in plasma and tumor based on the snap-PK (Supplementary Table 5) revealed that the optimal *in vitro* concentrations most likely are not achievable *in vivo* and this could explain the modest anti-proliferative effects found in the two *in vivo* mouse models. Also, healthy human PBMCs showed sensitivity toward AVA at doses of 3 *μ*M and 10 μM (Supplementary Figures 2b and c) that could explain the toxicity observed with doses >4 mg/kg in the mice. The reported CRM1 inhibitors have been shown to sensitize malignant cells to other cytotoxic agents,^53^ thus further supporting exploration of AVA in combination therapies.

In conclusion, we report functional and biologic activities of AVA, and an AVA analog in AML cells and *in vivo*. AVA and its existing analogs may thus represent a novel therapeutic strategy capable of targeting defined subsets of AML patients through their ability to interact with both NPM1 and CRM1, and possibly leukemia inititation cells as recently demonstrated for selinexor (KPT-330).^54^ We believe that further exploration of this family of compounds will lead to the identification of candidate molecules with improved physicochemical and PK properties for further evaluation in preclinical contexts.

## Materials and methods

### Cell lines and primary human samples

HL-60, NB4, MV4-11, Molm-13, HEK293, HCT-116 (ATCC, Manassas, VA, USA) and OCI-AML3 (DSMZ, Braunschweig, Germany) cells were grown according to the provider's instructions. Primary AML cells and CD34+ umbilical cord blood cells were cultured in StemSpan SFEM medium (Stem Cell Technologies Inc., Vancouver, BC, Canada).

The study was conducted in accordance with the Declaration of Helsinki and approved by the local Ethics Committee (Regional Ethics Committee West, University of Bergen, Bergen, Norway). Blood samples from consecutively diagnosed AML patients with high peripheral blood blast counts (>7 × 10^9^/l) were collected after informed consent and blood samples were processed by density gradient separation with >95% leukemic blasts for biobanking as previously described.^55^

Umbilical cord blood was collected from healthy newborns with subsequent CD34+ cell isolation from PBMCs using the CD34 MicroBead Kit (Miltenyi Biotec, Bergisch Gladbach, Germany).

PBMCs were acquired from random healthy blood donors at Department of Immunology and Transfusion Medicine, Haukeland University Hospital, Bergen, Norway.

### Reagents, antibodies and plasmids

AVA (C_26_H_27_N_3_O_4_, MW 445.51; AVA) and the BFA (C_29_H_29_N_3_O_3_, M.W. 467.56; BFA) were synthesized as previously described.^23, 26^

LMB was purchased from Sigma-Aldrich Corp. (St. Louis, MO, USA) and bortezomib from Millenium Pharmaceuticals (Cambridge, MA, USA).

NPM1 antibodies: anti-wt-NPM1^36^ (Invitrogen, Waltham, MA, USA, FC-61991); anti-NPM1mutantA (Abnova, Taipei City, Taiwan). The following antibodies were all purchased from Santa Cruz Biotechnology, Dallas, TX, USA: anti-CRM1 (H-300), anti-FLT3 (S-18), anti-p53 (Bp53-12) and anti-*β*-actin (sc-47778); from Abcam (Cambridge, UK): anti-RanBP1 (ab97659), anti-p21 (EA10) and anti-CoxIV (ab16056). Alexa-488/Alexa-568-goat-anti-mouse/rabbit (Life Technologies), POD-goat—anti-rabbit/mouse (Jackson ImmunoResearch), FITC-p53 (DO-7, BD Biosciences), Alexa-Fluor647-rabbit-anti-mouse/rabbit IgG(H+L) (Thermo Fisher Scientific, Waltham, MA, USA), PE-mouse-anti-human-CD45 (HI30, BD Biosciences, San Jose, CA, USA), PE-Cy7-anti-human-CD33 (P67.6, BD Biosciences), PE-mouse-anti-human-CD11b (D12, BD Biosciences), Biotin-anti-CD14 (M5E2, Biologend), Brilliant-Violet-605-anti-CD16 (3G8, Biolegend, San Diego, CA, USA), PE-anti-CD163 (MAC2,Trillium Diagnostic), Brilliant-Violet-711-anti-CD40 (2331,FUN-1, BD Biosciences), Pacific-Blue-anti-HLA-DR (MEM-12, EXBIO) and FITC-anti-CD45 (MEM-28, EXBIO, Praha, Czech Republic).

The NPM1wt-ECGFP plasmid (generous gift from Professor Marikki Laiho, Johns Hopkins University, USA) was used as template for PCR-based site-directed mutagenesis to generate the NPMc+-ECGFP plasmid with NPM1mutantA sequence.^14^ Both plasmids were verified by sequencing.

### Analysis of cell viability, proliferation and cell cycle

The WST-1 (Roche, Penzberg, Germany; measured by Spectramax Plus 384 Spectrophotometer, Molecular Devices Corporation, Sunnyvale CA, USA), resazurin (Life Technologies), Hoechst 3334, annexinV/propidium iodide (PI) staining (Nexins Research, Kattendijke, The Netherlands) assays and transmission electron microscopy were performed according to the manufacturer's protocol and as previously described.^56, 57^

### Transfection, cytospin, immunostaining and immunoblotting

OCI-AML3 cells were cytospun onto 12-mm coverslips using cytofunnel (400 r.p.m., 4 min; Shandon cytospin 3) Termo Fisher Scientific, Waltham, MA, USA. Transfection, fixation and immunostaining were performed as previously described.^58^ Cells were stained by May-Grünwald (Sigma-Aldrich)-Giemsa (Merck Milipore, Billerica, MA, USA) according to the manufacturer's protocol. Cells were analyzed by Zeiss Axio ObserverZ1 or Zeiss AxioVertA1 inverted microscopes and Carl Zeiss (Oberkochen, Germany) ZEN imaging software. Final figures were prepared using Adobe Photoshop CS5 (Adobe Systems Incorporated, San Jose, CA, USA). Immunoblotting was performed as previously described.^59^ All results are representative of at least three independent experiments.

### Phagocytosis, oxidative burst and NBT reduction assays

Phagocytosis and oxidative burst potential were measured in triplicates by flow cytometry using Beckman Coulter (Brea, CA, USA) EPICS XL-MCL as described previously.^60^

Briefly, *Staphylococcus aureus* Cowan III NCTC 8532 labeled with rhodamine green X (Molecular Probes, Waltham, MA, USA) were used as phagocytic targets while unlabeled bacteria were used as stimuli for oxidative burst measured with the substrate dihydrorhodamine 123 (Fluka, Buchs, Switzerland). Bacteria were not opsonized or opsonized with pooled human serum before both assays with PBMCs from a healthy individual as control. For the NBT reduction assay, cells 72 h post treatment were incubated with NBT (Sigma-Aldrich), stimulated with PMA (200 ng/ml) for 30 min at 37 °C, cytospun, counterstained with Safranin O (Sigma-Aldrich) before examined as described above.

### Flow cytometry analysis

Cells were fixed and processed and data was collected (FACS Calibur or Fortessa flow cytometer, BD Biosciences) and analyzed by FlowJo software (FlowJo LLC, Ashland, OR, USA) as previously described.^61^ For evaluation of myeloid differentiation cells were unfixed before staining.

### Mice

#### BALB/c nude mice

Conducted at Shanghai SLAC (shanghai laboratory animal center) laboratory Co. The experiments were performed according to international standards. Female BALB/c nude mice aged 7–8 weeks (18–20 g) were ordered and housed in specific pathogen-free conditions at ChemPartner (Shanghai, China). Animals were held for a minimum of 3 days for acclimation before beginning of the study.

#### NSG (NOD/SCID IL2r*γ*^null^) mice

The protocol for animal studies was approved by the Norwegian State Commission for Laboratory Animals and the experiments were performed according to the European Convention for the Protection of Vertebrates Used for Scientific Purposes. Female NSG mice (6–8 weeks old; Vivarium, University of Bergen) were maintained under defined flora conditions in individually ventilated cage that was kept on a 12 h dark/night schedule at a constant temperature of 21 °C and at 50% relative humidity. Bedding and cages were autoclaved and changed twice per month. The mice had continuous supply of sterile water and food, and were monitored daily by the same personnel for the duration of the experiment.

### Subcutaneous tumor models

BALB/c nude mice were injected subcutaneously with suspension of 5 × 10^6^ HTC-116 cells in serum-free medium (0.1 ml). Animals were monitored daily by general clinical observations throughout the study. Daily general health observation includes animal mortality, appearance, spontaneous activity, body posture, and food and water intake.

Tumor areas (length × width) were calculated by using digital callipers throughout the study period; tumor volumes were calculated based on the following formula: tumor volume=((length × width^2^)/2). The body weight of each mouse was measured every day before dosing and the tumor volume was measured twice per week. Animals showing signs of debilitations, marked body weight change with a 15% body weight loss (compared with body weight at start of study) for three consecutive days, or a 20% body weight loss at any time (>20%) and cachexia were humanely killed.

NSG mice were injected subcutaneously with 5 × 10^6^ OCI-AML3 cells in 0.1 ml sterile 1 × PBS with 10% Matrigel (BD Matrigel Basement Membrane Matrix, BD Biosciences) using a 28 G syringe. Animals were monitored closely for tumor growth; tumor volume (mm^3^)=(length × width × height × 3.14159265)/6) was measured twice weekly using digital callipers. Dosing started when tumors reached ~100 mm^3^; and then tumors were measured every third day. Animals were monitored daily by general clinical observations; weight (animals with >10% weight loss were humanely killed), ruffled fur, appearance, body posture, pale extremities, food and water intake. Vehicle control consisted of 10% DMSO in saline. When the tumors of the control animals reached 1000 mm^3^ all the animals were killed.

### Statistical analysis

Statistical comparisons were made using GraphPad Prism version 5.0. (La Jolla, CA, USA) One-way analysis of variance and Tukey's multiple comparison post tests were used to determine significant differences between several treatment groups. Unless otherwise stated, a Student's unpaired *t*-test was employed when only two groups were compared. IC_50_ values were calculated with curve fitting non-linear regression; Pearson or Spearman correlation determined significant dependency in Gaussian or nonparametric data, respectively. Normality in the data sets was determined using the D'Agostino and Pearson omnibus normality test. Data are presented as the mean ±S.E.M. of three independent experiments in triplicate unless otherwise stated. Differences with *P*<0.05 were considered statistically significant. Grubbs' method for assessing outliers was used on the *ex vivo* data before data analysis.

## Figures and Tables

**Table 1:** Molecular characteristics and IC_50_ values of AML cell lines

**Cell line**	**FAB**	**NPM1**	**FLT3**	**p53**	**IC50 (nM)**
NB4	M3	wt	wt	Mut	1100
HL-60	M2	wt	wt	Del	643
MV4-11	M5	wt	ITD	wt	116
OCI-AML3	M4	Mut A	wt	wt	112
Molm-13	M5	wt	ITD	wt	78

Abbreviations: Del, deletion; FAB, French-American-British classification; FLT3=fms-related tyrosine kinase 3; ITD, internal tandem duplication; Mut, mutant; wt, wild-type

## Supplementary Material

Supplementary Figure Legends

Supplementary Figure Legends Clean version

Supplementary Figure 4

Supplementary Tables Clean version

Supplementary Tables4

## References

[bib1] Estey E, Dohner H. Acute myeloid leukaemia. Lancet 2006; 368: 1894–1907. 17126723 10.1016/S0140-6736(06)69780-8

[bib2] Ferrara F, Schiffer CA. Acute myeloid leukaemia in adults. Lancet 2013; 381: 484–495. 23399072 10.1016/S0140-6736(12)61727-9

[bib3] Lowenberg B. Acute myeloid leukemia: the challenge of capturing disease variety. Hematology Am Soc Hematol Educ Program 2008: 1–11. 19074046 10.1182/asheducation-2008.1.1

[bib4] Borer RA, Lehner CF, Eppenberger HM, Nigg EA. Major nucleolar proteins shuttle between nucleus and cytoplasm. Cell 1989; 56: 379–390. 2914325 10.1016/0092-8674(89)90241-9

[bib5] Grisendi S, Mecucci C, Falini B, Pandolfi PP. Nucleophosmin and cancer. Nat Rev Cancer 2006; 6: 493–505. 16794633 10.1038/nrc1885

[bib6] Falini B, Bolli N, Liso A, Martelli MP, Mannucci R, Pileri S et al. Altered nucleophosmin transport in acute myeloid leukaemia with mutated NPM1: molecular basis and clinical implications. Leukemia 2009; 23: 1731–1743. 19516275 10.1038/leu.2009.124

[bib7] Wang W, Budhu A, Forgues M, Wang XW. Temporal and spatial control of nucleophosmin by the Ran-Crm1 complex in centrosome duplication. Nat Cell Biol 2005; 7: 823–830. 16041368 10.1038/ncb1282

[bib8] Burnett A, Wetzler M, Lowenberg B. Therapeutic advances in acute myeloid leukemia. J Clin Oncol 2011; 29: 487–494. 21220605 10.1200/JCO.2010.30.1820

[bib9] Cancer Genome Atlas Research N.. Genomic and epigenomic landscapes of adult de novo acute myeloid leukemia. N Engl J Med 2013; 368: 2059–2074. 23634996 10.1056/NEJMoa1301689 PMC3767041

[bib10] Dohner H, Estey EH, Amadori S, Appelbaum FR, Buchner T, Burnett AK et al. Diagnosis and management of acute myeloid leukemia in adults: recommendations from an international expert panel, on behalf of the European LeukemiaNet. Blood 2010; 115: 453–474. 19880497 10.1182/blood-2009-07-235358

[bib11] Martelli MP, Sportoletti P, Tiacci E, Martelli MF, Falini B. Mutational landscape of AML with normal cytogenetics: biological and clinical implications. Blood Rev 2013; 27: 13–22. 23261068 10.1016/j.blre.2012.11.001

[bib12] Schlenk RF, Dohner K, Krauter J, Frohling S, Corbacioglu A, Bullinger L et al. Mutations and treatment outcome in cytogenetically normal acute myeloid leukemia. N Engl J Med 2008; 358: 1909–1918. 18450602 10.1056/NEJMoa074306

[bib13] Dohner K, Schlenk RF, Habdank M, Scholl C, Rucker FG, Corbacioglu A et al. Mutant nucleophosmin (NPM1) predicts favorable prognosis in younger adults with acute myeloid leukemia and normal cytogenetics: interaction with other gene mutations. Blood 2005; 106: 3740–3746. 16051734 10.1182/blood-2005-05-2164

[bib14] Falini B, Mecucci C, Tiacci E, Alcalay M, Rosati R, Pasqualucci L et al. Cytoplasmic nucleophosmin in acute myelogenous leukemia with a normal karyotype. N Engl J Med 2005; 352: 254–266. 15659725 10.1056/NEJMoa041974

[bib15] Falini B, Nicoletti I, Martelli MF, Mecucci C. Acute myeloid leukemia carrying cytoplasmic/mutated nucleophosmin (NPMc+ AML): biologic and clinical features. Blood 2007; 109: 874–885. 17008539 10.1182/blood-2006-07-012252

[bib16] Lazenby M, Gilkes AF, Marrin C, Evans A, Hills RK, Burnett AK. The prognostic relevance of flt3 and npm1 mutations on older patients treated intensively or non-intensively: a study of 1312 patients in the UK NCRI AML16 trial. Leukemia 2014; 28: 1953–1959. 24573385 10.1038/leu.2014.90

[bib17] Falini B, Gionfriddo I, Cecchetti F, Ballanti S, Pettirossi V, Martelli MP. Acute myeloid leukemia with mutated nucleophosmin (NPM1): any hope for a targeted therapy? Blood Rev 2011; 25: 247–254. 21724308 10.1016/j.blre.2011.06.001

[bib18] Ivey A, Hills RK, Simpson MA, Jovanovic JV, Gilkes A, Grech A et al. Assessment of minimal residual disease in standard-risk AML. N Engl J Med 2016; 374: 422–433. 26789727 10.1056/NEJMoa1507471

[bib19] Alcalay M, Tiacci E, Bergomas R, Bigerna B, Venturini E, Minardi SP et al. Acute myeloid leukemia bearing cytoplasmic nucleophosmin (NPMc+ AML) shows a distinct gene expression profile characterized by up-regulation of genes involved in stem-cell maintenance. Blood 2005; 106: 899–902. 15831697 10.1182/blood-2005-02-0560

[bib20] Garzon R, Garofalo M, Martelli MP, Briesewitz R, Wang L, Fernandez-Cymering C et al. Distinctive microRNA signature of acute myeloid leukemia bearing cytoplasmic mutated nucleophosmin. Proc Natl Acad Sci USA 2008; 105: 3945–3950. 18308931 10.1073/pnas.0800135105 PMC2268779

[bib21] Schnittger S, Bacher U, Kern W, Alpermann T, Haferlach C, Haferlach T. Prognostic impact of FLT3-ITD load in NPM1 mutated acute myeloid leukemia. Leukemia 2011; 25: 1297–1304. 21537333 10.1038/leu.2011.97

[bib22] Thiede C, Steudel C, Mohr B, Schaich M, Schakel U, Platzbecker U et al. Analysis of FLT3-activating mutations in 979 patients with acute myelogenous leukemia: association with FAB subtypes and identification of subgroups with poor prognosis. Blood 2002; 99: 4326–4335. 12036858 10.1182/blood.v99.12.4326

[bib23] Myers AG, Herzon SB. Identification of a novel Michael acceptor group for the reversible addition of oxygen- and sulfur-based nucleophiles. Synthesis and reactivity of the 3-alkylidene-3H-indole 1-oxide function of avrainvillamide. J Am Chem Soc 2003; 125: 12080–12081. 14518979 10.1021/ja0372006

[bib24] Greshock TJ, Grubbs AW, Tsukamoto S, Williams RM. A concise, biomimetic total synthesis of stephacidin A and notoamide B. Angew Chem Int Ed Engl 2007; 46: 2262–2265. 17304610 10.1002/anie.200604378

[bib25] Mukherjee H, Chan KP, Andresen V, Hanley ML, Gjertsen BT, Myers AG. Interactions of the natural product (+)-avrainvillamide with nucleophosmin and exportin-1 mediate the cellular localization of nucleophosmin and its aml-associated mutants. ACS Chem Biol 2015; 10: 855–863. 25531824 10.1021/cb500872g PMC4652655

[bib26] Wulff JE, Siegrist R, Myers AG. The natural product avrainvillamide binds to the oncoprotein nucleophosmin. J Am Chem Soc 2007; 129: 14444–14451. 17958425 10.1021/ja075327f PMC2557551

[bib27] Calabrese EJ. Cancer biology and hormesis: human tumor cell lines commonly display hormetic (biphasic) dose responses. Crit Rev Toxicol 2005; 35: 463–582. 16422392 10.1080/10408440591034502

[bib28] Stapnes C, Ryningen A, Hatfield K, Oyan AM, Eide GE, Corbascio M et al. Functional characteristics and gene expression profiles of primary acute myeloid leukaemia cells identify patient subgroups that differ in susceptibility to histone deacetylase inhibitors. Int J Oncol 2007; 31: 1529–1538. 17982680

[bib29] Lowenberg B, van Putten WL, Touw IP, Delwel R, Santini V. Autonomous proliferation of leukemic cells *in vitro* as a determinant of prognosis in adult acute myeloid leukemia. N Engl J Med 1993; 328: 614–619. 8429853 10.1056/NEJM199303043280904

[bib30] Thiede C, Koch S, Creutzig E, Steudel C, Illmer T, Schaich M et al. Prevalence and prognostic impact of NPM1 mutations in 1485 adult patients with acute myeloid leukemia (AML). Blood 2006; 107: 4011–4020. 16455956 10.1182/blood-2005-08-3167

[bib31] Quentmeier H, Martelli MP, Dirks WG, Bolli N, Liso A, Macleod RA et al. Cell line OCI/AML3 bears exon-12 NPM gene mutation-A and cytoplasmic expression of nucleophosmin. Leukemia 2005; 19: 1760–1767. 16079892 10.1038/sj.leu.2403899

[bib32] Anensen N, Hjelle SM, Van Belle W, Haaland I, Silden E, Bourdon JC et al. Correlation analysis of p53 protein isoforms with NPM1/FLT3 mutations and therapy response in acute myeloid leukemia. Oncogene 2012; 31: 1533–1545. 21860418 10.1038/onc.2011.348

[bib33] Cavalcanti GB Jr., Scheiner MA, Simoes Magluta EP, Vasconcelos FC, Klumb CE, Maia RC. p53 flow cytometry evaluation in leukemias: correlation to factors affecting clinical outcome. Cytometry B Clin Cytom 2010; 78: 253–259. 20198607 10.1002/cyto.b.20514

[bib34] Rucker FG, Schlenk RF, Bullinger L, Kayser S, Teleanu V, Kett H et al. TP53 alterations in acute myeloid leukemia with complex karyotype correlate with specific copy number alterations, monosomal karyotype, and dismal outcome. Blood 2012; 119: 2114–2121. 22186996 10.1182/blood-2011-08-375758

[bib35] McCormack E, Haaland I, Venas G, Forthun RB, Huseby S, Gausdal G et al. Synergistic induction of p53 mediated apoptosis by valproic acid and nutlin-3 in acute myeloid leukemia. Leukemia 2012; 26: 910–917. 22064349 10.1038/leu.2011.315

[bib36] Falini B, Bolli N, Shan J, Martelli MP, Liso A, Pucciarini A et al. Both carboxy-terminus NES motif and mutated tryptophan(s) are crucial for aberrant nuclear export of nucleophosmin leukemic mutants in NPMc+ AML. Blood 2006; 107: 4514–4523. 16455950 10.1182/blood-2005-11-4745

[bib37] Pasqualucci L, Liso A, Martelli MP, Bolli N, Pacini R, Tabarrini A et al. Mutated nucleophosmin detects clonal multilineage involvement in acute myeloid leukemia: Impact on WHO classification. Blood 2006; 108: 4146–4155. 16926285 10.1182/blood-2006-06-026716

[bib38] Boulon S, Westman BJ, Hutten S, Boisvert FM, Lamond AI. The nucleolus under stress. Mol Cell 2010; 40: 216–227. 20965417 10.1016/j.molcel.2010.09.024 PMC2987465

[bib39] Ranganathan P, Yu X, Na C, Santhanam R, Shacham S, Kauffman M et al. Preclinical activity of a novel CRM1 inhibitor in acute myeloid leukemia. Blood 2012; 120: 1765–1773. 22677130 10.1182/blood-2012-04-423160 PMC3433086

[bib40] Balusu R, Fiskus W, Rao R, Chong DG, Nalluri S, Mudunuru U et al. Targeting levels or oligomerization of nucleophosmin 1 induces differentiation and loss of survival of human AML cells with mutant NPM1. Blood 2011; 118: 3096–3106. 21719597 10.1182/blood-2010-09-309674 PMC6710561

[bib41] McCormack E, Mujic M, Osdal T, Bruserud O, Gjertsen BT. Multiplexed mAbs: a new strategy in preclinical time-domain imaging of acute myeloid leukemia. Blood 2013; 121: e34–e42. 23243270 10.1182/blood-2012-05-429555

[bib42] Levine AJ, Hu W, Feng Z. The P53 pathway: what questions remain to be explored? Cell Death Differ 2006; 13: 1027–1036. 16557269 10.1038/sj.cdd.4401910

[bib43] Hou HA, Chou WC, Kuo YY, Liu CY, Lin LI, Tseng MH et al. TP53 mutations in de novo acute myeloid leukemia patients: longitudinal follow-ups show the mutation is stable during disease evolution. Blood Cancer J 2015; 5: e331. 26230955 10.1038/bcj.2015.59 PMC4526785

[bib44] Seifert H, Mohr B, Thiede C, Oelschlagel U, Schakel U, Illmer T et al. The prognostic impact of 17p (p53) deletion in 2272 adults with acute myeloid leukemia. Leukemia 2009; 23: 656–663. 19151774 10.1038/leu.2008.375

[bib45] Suzuki T, Kiyoi H, Ozeki K, Tomita A, Yamaji S, Suzuki R et al. Clinical characteristics and prognostic implications of NPM1 mutations in acute myeloid leukemia. Blood 2005; 106: 2854–2861. 15994285 10.1182/blood-2005-04-1733

[bib46] Hutten S, Kehlenbach RH. CRM1-mediated nuclear export: to the pore and beyond. Trends Cell Biol 2007; 17: 193–201. 17317185 10.1016/j.tcb.2007.02.003

[bib47] Sakakibara K, Saito N, Sato T, Suzuki A, Hasegawa Y, Friedman JM et al. CBS9106 is a novel reversible oral CRM1 inhibitor with CRM1 degrading activity. Blood 2011; 118: 3922–3931. 21841164 10.1182/blood-2011-01-333138

[bib48] Etchin J, Sanda T, Mansour MR, Kentsis A, Montero J, Le BT et al. KPT-330 inhibitor of CRM1 (XPO1)-mediated nuclear export has selective anti-leukaemic activity in preclinical models of T-cell acute lymphoblastic leukaemia and acute myeloid leukaemia. Br J Haematol 2013; 161: 117–127. 23373539 10.1111/bjh.12231 PMC3980736

[bib49] Kojima K, Kornblau SM, Ruvolo V, Dilip A, Duvvuri S, Davis RE et al. Prognostic impact and targeting of CRM1 in acute myeloid leukemia. Blood 2013; 121: 4166–4174. 23564911 10.1182/blood-2012-08-447581 PMC3656451

[bib50] Lapalombella R, Sun Q, Williams K, Tangeman L, Jha S, Zhong Y et al. Selective inhibitors of nuclear export show that CRM1/XPO1 is a target in chronic lymphocytic leukemia. Blood 2012; 120: 4621–4634. 23034282 10.1182/blood-2012-05-429506 PMC3512237

[bib51] Zhang K, Wang M, Tamayo AT, Shacham S, Kauffman M, Lee J et al. Novel selective inhibitors of nuclear export CRM1 antagonists for therapy in mantle cell lymphoma. Exp Hematol 2013; 41: 67–78 e64. 22986101 10.1016/j.exphem.2012.09.002

[bib52] Johnson DE, Redner RL. An ATRActive future for differentiation therapy in AML. Blood Rev 2015; 29: 263–268. 25631637 10.1016/j.blre.2015.01.002 PMC4494875

[bib53] Turner JG, Dawson J, Cubitt CL, Baz R, Sullivan DM. Inhibition of CRM1-dependent nuclear export sensitizes malignant cells to cytotoxic and targeted agents. Semin Cancer Biol 2014; 27: 62–73. 24631834 10.1016/j.semcancer.2014.03.001 PMC4108511

[bib54] Etchin J, Montero J, Berezovskaya A, Le BT, Kentsis A, Christie AL et al. Activity of a selective inhibitor of nuclear export, selinexor (KPT-330), against AML-initiating cells engrafted into immunosuppressed NSG mice. Leukemia 2016; 30: 190–199. 26202935 10.1038/leu.2015.194 PMC4994896

[bib55] Bruserud O, Gjertsen BT, Foss B, Huang TS. New strategies in the treatment of acute myelogenous leukemia (AML): *in vitro* culture of aml cells—the present use in experimental studies and the possible importance for future therapeutic approaches. Stem Cells 2001; 19: 1–11. 11209086 10.1634/stemcells.19-1-1

[bib56] Bredholt T, Ersvaer E, Erikstein BS, Sulen A, Reikvam H, Aarstad HJ et al. Distinct single cell signal transduction signatures in leukocyte subsets stimulated with khat extract, amphetamine-like cathinone, cathine or norephedrine. BMC Pharmacol Toxicol 2013; 14: 35. 23845085 10.1186/2050-6511-14-35 PMC3733921

[bib57] Erikstein BS, Hagland HR, Nikolaisen J, Kulawiec M, Singh KK, Gjertsen BT et al. Cellular stress induced by resazurin leads to autophagy and cell death via production of reactive oxygen species and mitochondrial impairment. J Cell Biochem 2010; 111: 574–584. 20568117 10.1002/jcb.22741 PMC2946440

[bib58] Andresen V, Pise-Masison CA, Sinha-Datta U, Bellon M, Valeri V, Washington Parks R et al. Suppression of HTLV-1 replication by Tax-mediated rerouting of the p13 viral protein to nuclear speckles. Blood 2011; 118: 1549–1559. 21677314 10.1182/blood-2010-06-293340 PMC3156045

[bib59] Silden E, Hjelle SM, Wergeland L, Sulen A, Andresen V, Bourdon JC et al. Expression of TP53 isoforms p53beta or p53gamma enhances chemosensitivity in TP53(null) cell lines. PLoS One 2013; 8: e56276. 23409163 10.1371/journal.pone.0056276 PMC3569410

[bib60] Kielland LG, Vage RA, Eide GE, Sornes S, Naess A. Anti-tuberculosis drugs and human polymorphonuclear leukocyte functions. Chemotherapy 2011; 57: 339–344. 21912116 10.1159/000330442

[bib61] Skavland J, Jorgensen KM, Hadziavdic K, Hovland R, Jonassen I, Bruserud O et al. Specific cellular signal-transduction responses to *in vivo* combination therapy with ATRA, valproic acid and theophylline in acute myeloid leukemia. Blood Cancer J 2011; 1: e4. 22829110 10.1038/bcj.2011.2 PMC3255270

