# Supplementary material for: Anti-proliferative activity of the NPM1 interacting natural product avrainvillamide in acute myeloid leukemia
Source: Cell Death Dis. 2016 Dec 1;7(12):e2497–. doi: 10.1038/cddis.2016.392 (PMC5260983; doi:10.1038/cddis.2016.392)
Supplement: Supplementary Figure Legends [file cddis2016392x1.pdf]

SUPPLEMENTARY FIGURES LEGENDS

Supplementary Figure 1. (**a**) Histogram of MV4-11 cells incubated with DMSO or 0.3µM avrainvillamide for 24h, followed by PI staining and analysis by flow cytometry. Representative result is shown. (**b**) MV4-11 cells were treated with 30nM, 100nM or 300nM avrainvillamide for 24h prior to PI staining and analysis by flow cytometry. Results represent the mean ±SEM of the relative values compared to the untreated controls in SubG1, G1, S and G_2/M phase (*n*=3; **P* < 0.05, ****P* < 0.001).

Supplementary Figure 2. (**a**) Primary AML cells were incubated with avrainvillamide (10nM to 30µM) for 24h and proliferation was assayed by ^{3}H-thymidine incorporation where the mean ±SEM of the values relative to vehicle controls are presented. (**b**) PBMCs from 5 healthy blood donors were incubated for 24h with DMSO (CTR) and avrainvillamide (0.05, 0.1, 1, 3 and 10µM), stained with annexinV-Alexa647/PI and analyzed by flow cytometry. Representative histograms for CTR (DMSO), 1, 3 and 10 µM avrainvillamide. Viable cells represent annexinV-PI negative cells. (**c**) AnnexinV-PI negative cells (live cells) normalized to CTR displayed against concentration of avrainvillamide.

Supplementary Figure ~~1~~3. (**a**) ~~Primary AML cells were incubated with avrainvillamide (10nM to 30µM) for 24h and proliferation was assayed by ^{3}H-thymidine incorporation where the mean ±SEM of the values relative to vehicle controls are presented. (**b**)~~ AML patient samples with FAB subtype M1 (*n=15*) and AML patient samples with FAB subtype M5 (*n=13*) were treated

with avrainvillamide (10µM) for 24h and measured with the WST-1 assay. Results represent the mean ±SEM of the values relative to vehicle controls (**P<0.01). (**b**~~c~~-**d**~~e~~) Multiparameter flow cytometry analysis of the expression of CD15 (**b**~~c~~), CD14 (**c**~~d~~) and CD11c (**d**~~e~~) plotted against cellular sensitivity towards avrainvillamide (10µM, 24h) as measured by the WST-1 assay. Results represent mean ±SEM of values relative to vehicle controls. Circles represent mean of the relative values compared to the untreated controls (CD15; *P<0.05; CD14; *P<0.05, CD11c: Pearson's correlation: R^2 = 0.17, P<0.05). (~~**f**~~**e**) Primary AML patient samples were incubated with avrainvillamide (10µM) for 24h. Circles represent means of the values relative to vehicle controls, plotted against cellular proliferation rate as determined by ^{3}H-thymidine incorporation in control samples (Pearson's correlation: R^2= 0.25, P<0.01).

Supplementary Figure ~~2~~4. Representative histograms of OCI-AML3 cells treated for 72h with DMSO (CTR), AVA (0.5µM), BFA (0.5µM) or KPT-330 (0.1µM). Live cells were stained with antibodies (CD163, CD86, CD14 and CD11b) and analyzed by flow cytometry for expression by gating on living cells (dead cells excluded by PI or TO-PRO3 stain). Stained and unstained cells are represented as solid and dotted lines, respectively.

~~**Supplementary Figure 3.** The effect of avrainvillamide on healthy PBMCs. **(a)** PBMCs from 5 healthy blood donors were incubated for 24h with DMSO (CTR) and avrainvillamide (0.05µM, 0.1µM, 1µM, 3µM and 10µM), stained with annexin V-Alexa647/PI and analyzed by flow cytometry. Annexin V negative cells (live cells) normalized to CTR displayed against concentration of avrainvillamide. (**b**) Representative histogram for 0 (CTR), 1, 3 and 10 µM avrainvillamide. Viable cells represent annexin V negative cells.~~
