# Supplementary figures and images for: Anti-proliferative activity of the NPM1 interacting natural product avrainvillamide in acute myeloid leukemia
Source: Cell Death Dis. 2016 Dec 1;7(12):e2497–. doi: 10.1038/cddis.2016.392 (PMC5260983; doi:10.1038/cddis.2016.392)

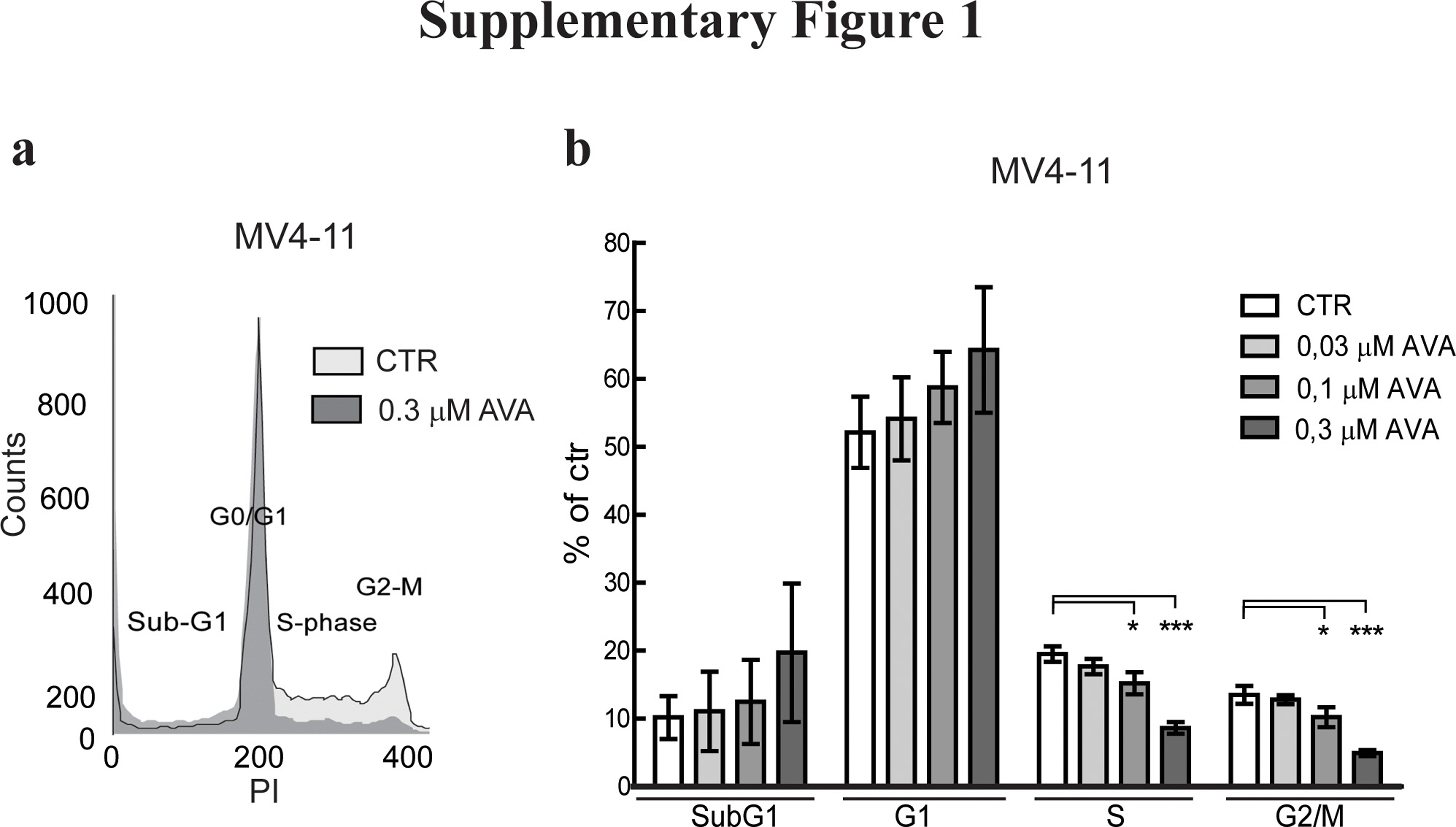

Supplement: Supplementary Figure 1 [file cddis2016392x3.tif]

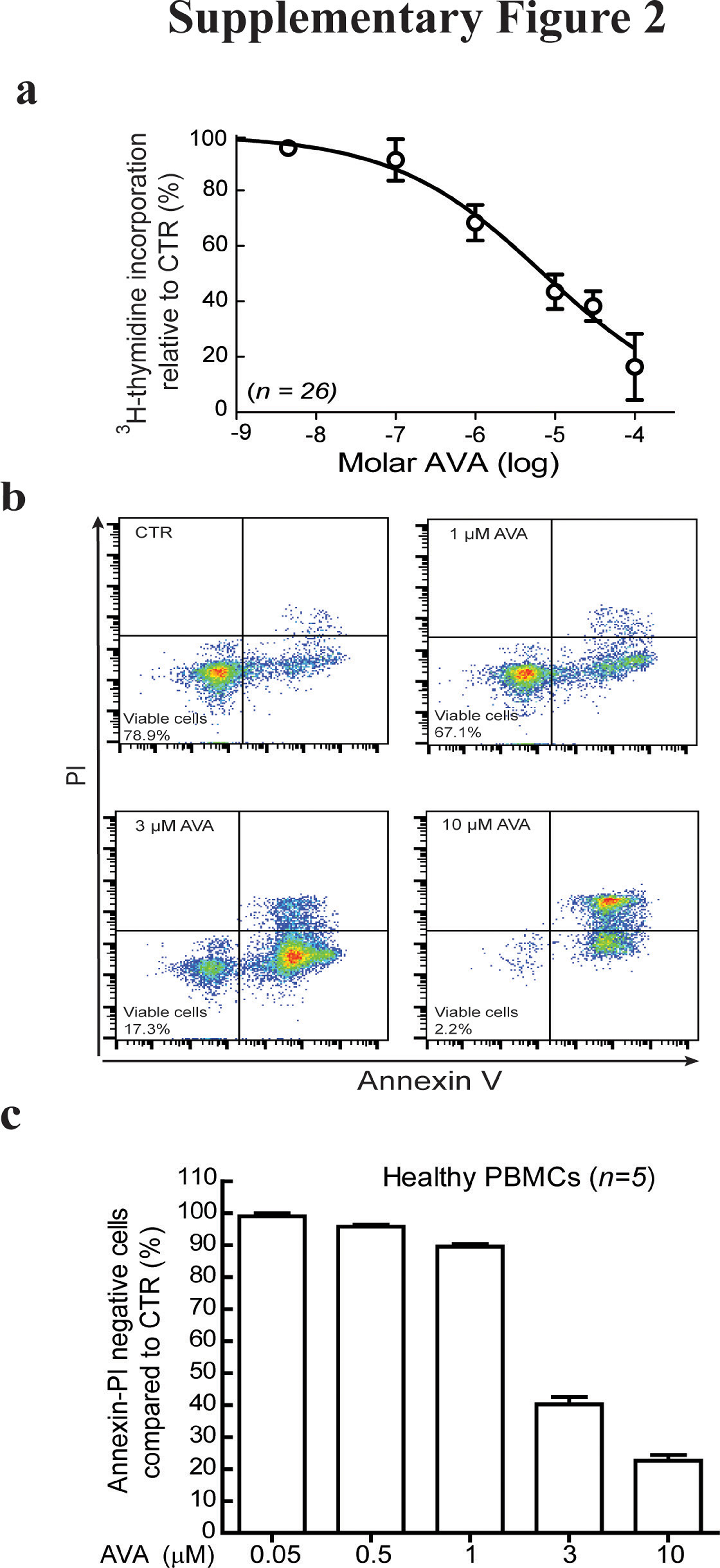

Supplement: Supplementary Figure 2 [file cddis2016392x4.tif]

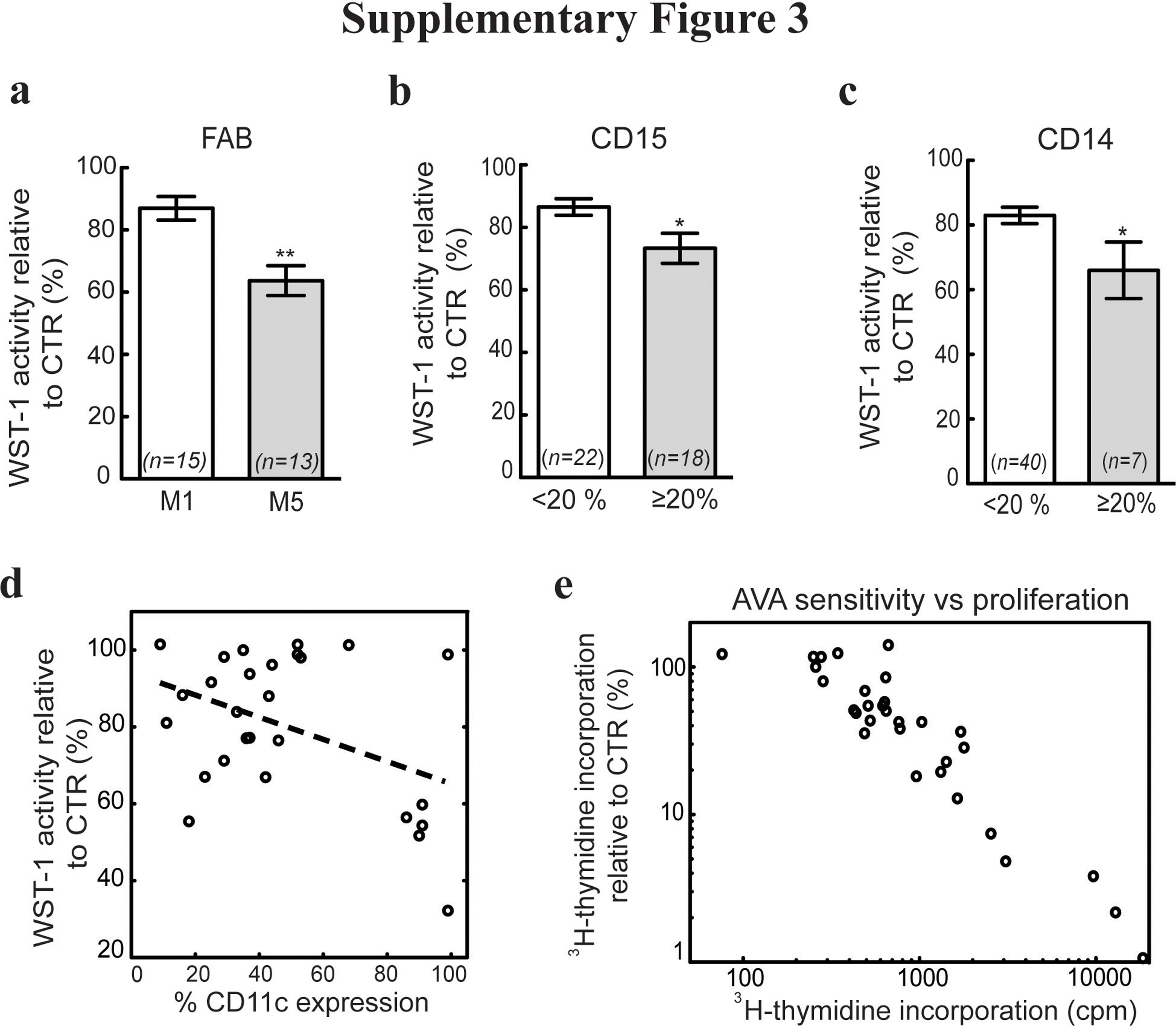

Supplement: Supplementary Figure 3 [file cddis2016392x5.tif]
