# Supplementary material for: Anti-proliferative activity of the NPM1 interacting natural product avrainvillamide in acute myeloid leukemia
Source: Cell Death Dis. 2016 Dec 1;7(12):e2497–. doi: 10.1038/cddis.2016.392 (PMC5260983; doi:10.1038/cddis.2016.392)
Supplement: Supplementary Tables Clean version [file cddis2016392x7.docx]

| **Supplementary Table 1. Characteristics of primary AML patient samples; 24h assay.** | | | | | | | |
| --- | --- | --- | --- | --- | --- | --- | --- |
| **Patient**  **no.** | **Age** | **Sex** | **FAB** | **Cytogenetics** | **FLT3** | **NPM1**  **(type mutation)** | **Previous**  **disease** |
| 1 | 24 | M | M2 | Complex | wt | wt | CML |
| 2 | 58 | M | M5 | 46,XY | wt | wt | *De novo* |
| 3 | 77 | F | M1 | nt | ITD | wt | *De novo* |
| 4 | 62 | M | M2 | t(4;20) | wt | wt | Relapse |
| 5 | 86 | F | M1 | 46,XX | nt | nt | *De novo* |
| 6 | 65 | M | M5 | Complex | wt | Ins TCTG(A) | *De novo* |
| 7 | 29 | F | M5 | 46,XX | ITD, asp835 | wt | *De novo* |
| 8 | 61 | M | M2 | -7 | wt | Ins CCTG(D) | Relapse |
| 9 | 53 | M | M0 | +13 | wt | wt | *De novo* |
| 10 | 60 | F | M5 | 46,XX | ITD | Ins TCTG (A) | *De novo* |
| 11 | 72 | M | M1 | Complex | wt | wt | MDS |
| 12 | 60 | F | M1 | 46,XX | wt | Ins TCTG(A) | *De novo* |
| 13 | 67 | F | M5 | t(9;11), +19 | wt | wt | *De novo* |
| 14 | 68 | F | M1 | 46,XX | ITD | wt | Relapse |
| 15 | 68 | M | M4 | 46, XY | asp835 | wt | MF |
| 16 | 53 | M | M5 | nt | wt | Ins TCTG(A) | *De novo* |
| 17 | 79 | M | M5 | nt | wt | wt | *De novo* |
| 18 | 75 | F | M4 | 46,XX | ITD | wt | *De novo* |
| 19 | 45 | F | M4 | 46,XX | wt | Ins TAGG | *De novo* |
| 20 | 75 | F | M1 | nt | ITD | wt | *De novo* |
| 21 | 82 | M | M0 | nt | wt | wt | PV |
| 22 | 63 | F | M4 | 46,XX | ITD | wt | *De novo* |
| 23 | 76 | M | M0 | 46,XY | wt | wt | *De novo* |
| 24 | 46 | M | M4 | 46,XY | ITD | Ins TGCA | Relapse |
| 25 | 32 | F | M5 | del(5q) | wt | Ins TCTG(A) | *De novo* |
| 26 | 29 | M | M4 | 46,XY | ITD | Ins TCTG(A) | Relapse |
| 27 | 62 | M | M4 | +8 | wt | nt | *De novo* |
| 28 | 68 | M | M2 | Complex | wt | wt | *De novo* |
| 29 | 59 | F | M2 | -7 | wt | wt | *De novo* |
| 30 | 82 | M | M2 | nt | wt | wt | *De novo* |
| 31 | 49 | F | M2 | Complex | wt | wt | CML +  relapse |
| 32 | 82 | M | M5 | - Y | wt | wt | *De novo* |
| 33 | 81 | F | M1 | 46,XX | wt | wt | MDS |
| 34 | 33 | M | M1 | 46,XY | wt | wt | *De novo* |
| 35 | 35 | M | M2 | 46,XY | wt | wt | *De novo* |
| 36 | 48 | M | M4 | inv(16) | wt | wt | *De novo* |
| 37 | 59 | F | M4 | 46,XX | ITD | Ins TCTG(A) | Relapse |
| 38 | 36 | M | M5 | inv(16),  +8, +22 | ITD | wt | *De novo* |
| 39 | 70 | F | M4 | 46,XX | wt | Ins TGCA | *De novo* |
| 40 | 60 | M | M4 | 46,XY | ITD | wt | *De novo* |
| 41 | 84 | M | M1 | Complex | wt | wt | *De novo* |
| 42 | 59 | F | M4 | 46,XX | ITD | Ins | *De novo* |
| 43 | 72 | F | M1 | nt | nt | nt | *De novo* |

FAB = French-American-British classification; FLT3 = fms-related tyrosine kinas 3; wt = wild type; mut = mutant; Ins = insertion; MDS = myelodysplastic syndrome; nt = not determined; PV = polycythemia vera; CML = chronic myeloid leukemia.

| **Supplementary Table 2. IC50 values of primary AML patient samples** | | | | | | | | | | |
| --- | --- | --- | --- | --- | --- | --- | --- | --- | --- | --- |
| **Patient**  **No.** | **FAB** | **Cytogenetics** | | **FLT3** | **NPM1** | | **Previous IC50, μM**  **disease** | | |  |
| 1 | M2 | | Complex | wt wt CML | | | |  | 0.5 | |
| 2 | M5 | | 46,XY wt | | wt | *De novo* | | | 3.6 | |
| 3 | M1 | | nt ITD | | wt | *De novo* | | | 0.7 | |
| 5 | M1 | | 46,XX nt | | nt | *De novo* | | | 27.3 | |
| 7 | M5 | | Complex wt | | Ins | *De novo* | | | 19.4 | |
| 8 | M2 | | -7 wt | | Ins | *De novo* | | | 110.3 (res) | |
| 10 | M5 | | 46,XX ITD | | Ins | *De novo* | | | 6.4 | |
| 11 | M1 | | Complex wt | | wt | MDS | | | 0.6 | |
| 13 | M5 | | t(9:11),+19 wt | | wt | *De novo* | | | 1.1 | |
| 18 | M4 | | 46,XX ITD | | wt | *De novo* | | | 27.9 | |
| 19 | M4 | | 46,XX wt | | Ins | *De novo* | | | 7.5 | |
| 24 | M4 | | 46,XY ITD | | Ins | Relapse | | | 1.4 | |
| 25 | M5 | | del(5q) wt | | Ins | *De novo* | | | 788 (res) | |
| 26 | M4 | | 46,XY ITD | | Ins | Relapse | | | 207 (res) | |
| 27 | M4 | | +8 wt | | nt | *De novo* | | | 0.8 | |
| 28 | M2 | | Complex wt | | wt | *De novo* | | | 5.8 | |
| 30 | M2 | | nt wt | | wt | *De novo* | | | 53.7 | |
| 31 | M2 | | Complex wt | | wt | CML | | | 3.1 | |
| 32 | M5 | | -Y wt | | wt | *De novo* | | | 17.1 | |
| 33 | M1 | | 46,XX wt | | wt | MDS | | | 11.1 | |
| 36 | M4 | | inv(16) wt | | wt | *De novo* | | | 1.8 | |
| 41 | M1 | | Complex wt | | wt | *De novo* | | | 0.5 | |
| 42 | M4 | | 46,XX ITD | | Ins | *De novo* | | | 0.3 | |
| 43 | M1 | | nt nt | | nt | *De novo* | | | 71340(res) | |

FAB = French-American-British classification; FLT3 = fms-related tyrosine kinas 3; wt = wild type; mut = mutant; Ins = insertion; MDS = myelodysplastic syndrome; nt = not determined; CML = chronic myeloid leukemia; res = resistant.

| **Supplementary Table 3. Characteristics of AML patient samples; 7 days assay with cytokines.** | | | | | | | |
| --- | --- | --- | --- | --- | --- | --- | --- |
| **Patient**  **no.** | **Age** | **Sex** | **FAB** | **Cytogenetics** | **FLT3** | **NPM-1**  **(type mutation)** | **Previous**  **disease** |
| 1 | 24 | M | M2 | Complex | wt | wt | CML |
| 2 | 58 | M | M5 | 46,XY | wt | wt | *De novo* |
| 3 | 77 | F | M1 | nt | ITD | wt | *De novo* |
| 6 | 65 | M | M5 | Complex | wt | Ins TCTG(A) | *De novo* |
| **7** | 29 | F | M5 | 46,XX | ITD,Asp835 | wt | *De novo* |
| 10 | 60 | F | M5 | 46,XX | ITD | Ins TCTG(A) | *De novo* |
| 13 | 67 | F | M5 | t(9:11),+19 | wt | wt | *De novo* |
| **17** | 79 | M | M5 | nt | wt | wt | *De novo* |
| 18 | 75 | F | M4 | 46,XX | ITD | wt | *De novo* |
| 19 | 45 | F | M4 | 46,XX | wt | Ins TAGG | *De novo* |
| 20 | 75 | F | M1 | nt | ITD | wt | *De novo* |
| 23 | 76 | M | M0 | 46,XY | wt | wt | *De novo* |
| 25 | 32 | F | M5 | del(5q) | wt | Ins | *De novo* |
| 29 | 59 | F | M2 | -7 | wt | wt | *De novo* |
| 30 | 82 | M | M2 | nt | wt | wt | *De novo* |
| 31 | 49 | F | M2 | Complex | wt | wt | CML+relapse |
| 33 | 81 | F | M1 | 46,XX | wt | nt | MDS |
| 34 | 33 | M | M1 | 46,XY | wt | wt | *De novo* |
| 35 | 35 | M | M2 | 46,XY | wt | wt | *De novo* |
| 36 | 48 | M | M4 | inv(16) | wt | wt | *De novo* |
| 37 | 59 | F | M4 | 46,XX | ITD | Ins | *De novo* |
| 38 | 36 | M | M5 | inv(16), +8, +22 | ITD | wt | *De novo* |
| 39 | 70 | F | M4 | 46,XX | wt | Ins TGCA | *De novo* |
| 39 | 70 | F | M4 | nt | wt | Ins TCTG(A) | MDS |
| 40 | 60 | M | M4 | 46,XY | ITD | wt | *De novo* |
| 41 | 84 | M | M1 | Complex | wt | wt | *De novo* |
| 42 | 59 | F | M4 | 46,XX | ITD | Ins | *De novo* |
| 43 | 72 | F | M1 | nt | nt | nt | *De novo* |
| 44 | 67 | M | M0 | del(5q) | ITD | wt | *De novo* |
| 45 | 71 | M | M2 | 46,XY | asp835 | Ins TCTG(A)/CCTG(D) | Relapse |
| 46 | 72 | M | M5 | 46,XY | wt | Ins TCTG(A) | *De novo* |
| 47 | 43 | M | M1 | 46,XY | wt | wt | *De novo* |
| 48 | 64 | F | M2 | 46,XX | ITD | Ins | Relapse |
| 49 | 72 | M | M4 | 46,XY | ITD | wt | *De novo* |
| 50 | 36 | M | M5 | Complex | ITD | wt | *De novo* |
| 51 | 81 | F | M2 | nt | ITD | Ins TCTG(A) | *De novo* |
| 52 | 42 | F | M5 | 46,XX | wt | Ins TCTG(A) | *De novo* |
| 53 | 65 | M | M4 | 46,XY | wt | wt | *De novo* |
| 54 | 78 | F | M0 | Complex | wt | wt | *De novo* |
| 55 | 80 | F | M2 | Complex | wt | wt | *De novo* |
| 56 | 46 | M | M1 | 46,XY | wt | Ins TCTG(A) | *De novo* |
| 57 | 74 | F | M2 | nt | wt | wt | *De novo* |
| 58 | 65 | M | M5 | 46,XY | ITD | Ins TCTG(A) | *De novo* |
| 59 | 68 | M | M2 | nt | ITD | Ins | MDS |
| 60 | 82 | F | M4 | nt | ITD | wt | *De novo* |
| 61 | 46 | F | M1 | inv(16) | wt | wt | *De novo* |
| 62 | 56 | F | M1 | 46,XX | wt | wt | *De novo* |
| 63 | 64 | M | M1 | nt | ITD | Ins TCTG(A) | *De novo* |
| 64 | 44 | F | M1 | del(7q) | ITD | wt | *De novo* |
| 65 | 66 | F | M1 | 46,XX | wt | Ins | *De novo* |
| 66 | 67 | M | M1 | 46,XY | ITD | wt | Relapse |
| 67 | 61 | M | M4 | 46,XY | ITD | Ins TCAG | *De novo* |
|  |  |  |  |  |  |  |  |

FAB = French-American-British classification; FLT3 = fms-related tyrosine kinas 3; wt = wild type; mut = mutant; Ins = insertion; MDS = myelodysplastic syndrome; nt = not determined; CML = chronic myeloid leukemia.

| **Supplementary Table 4. Characteristics of primary AML patient samples** | | | | | | | |
| --- | --- | --- | --- | --- | --- | --- | --- |
| **Patient**  **no.** | **Age** | **S** | **FAB** | **Cytogenetics** | **FLT3** | **NPM1**  **(Type mutation)** | **Previous**  **disease** |

| 46 | 72 | M | M5 | 46,XY | wt | Ins TCTG(A) | *De novo* |
| --- | --- | --- | --- | --- | --- | --- | --- |
| 56 | 46 | M | M1 | 46,XY | wt | Ins TCTG(A) | *De novo* |
| 68 | 64 | M | M5 | 46,XY | wt | Ins TCTG(A) | *De novo* |
| 69 | 68 | F | M5 | 46,XX | wt | Ins TCTG(A) | *De novo* |

FAB = French-American-British classification; FLT3 = fms-related tyrosine kinase 3; wt = wild type; Ins = insertion.

| **Supplementary Table 5. Concentration of BFA in plasma and tumor.** | | |
| --- | --- | --- |
| **Time (h)** | **Plasma (nM)**  **1 2 Mean** | **Tumor (nM)**  **1 2 Mean** |
| 0 | 0,00 0,00 0,00 | 0,00 0,00 0,00 |
| 1 | 181,37 145,22 163,40 | 57,53 81,49 69,51 |
| 6 | 61.81 71,86 66,94 | 30,58 42,77 37,21 |
| 24 | 13,92 9,52 11,76 | 16,68 21,11 18,80 |

Snap-PK for 2 mg/kg BFA (Figure 7A) in two BALB/c mice (1,2), BFA: M.W. 467.56.
