# Supplementary material for: Anti-proliferative activity of the NPM1 interacting natural product avrainvillamide in acute myeloid leukemia
Source: Cell Death Dis. 2016 Dec 1;7(12):e2497–. doi: 10.1038/cddis.2016.392 (PMC5260983; doi:10.1038/cddis.2016.392)
Supplement: Supplementary Tables4 [file cddis2016392x8.pdf]

Supplementary Table 1. Cell cycle analysis of the effects of avrainvillamide on Molm-13 cells.

Avrainvillamide (µM)	Untreated		0.3		1	
	%	AU	%	AU	%	AU
**G0/G1**	100	41	137	56	115	47
**G2-M**	100	24	71	17	42	10
**Synthesis**	100	28	61	19	32	9
**Proliferation (^{3}H-Thy)**	100	64000 cpm	45	29000 cpm	4	2600 cpm
**Sub-G1**	100	4	150	6	725	29
**Apoptosis (Hoechst)**	6	18/300	12	36/300	72	216/300

Molm-13 cells were incubated with DMSO or avrainvillamide (0.3 or 1µM) for 24h. Results reported as percent relative to vehicle controls in arbitrary units (AU). AU reported in the cell cycle results for untreated cells indicate percentages of the total number of cells assesed with PI (*n* = 3-5) (cpm = counts per minute).

Supplementary Table ~~2~~1. Characteristics of primary AML patient samples; 24h assay.

Patient no.	Age	Sex	FAB	Cytogenetics	FLT3	NPM1 (type mutation)	Previous disease
1	24	M	M2	Complex	wt	wt	CML
2	58	M	M5	46,XY	wt	wt	*De novo*
3	77	F	M1	nt	ITD	wt	*De novo*
4	62	M	M2	t(4;20)	wt	wt	Relapse
5	86	F	M1	46,XX	nt	nt	*De novo*
6	65	M	M5	Complex	wt	Ins TCTG(A)	*De novo*
7	29	F	M5	46,XX	ITD, asp835	wt	*De novo*
8	61	M	M2	-7	wt	Ins CCTG(D)	Relapse
9	53	M	M0	+13	wt	wt	*De novo*
10	60	F	M5	46,XX	ITD	Ins TCTG (A)	*De novo*
11	72	M	M1	Complex	wt	wt	MDS
12	60	F	M1	46,XX	wt	Ins TCTG(A)	*De novo*
13	67	F	M5	t(9;11), +19	wt	wt	*De novo*
14	68	F	M1	46,XX	ITD	wt	Relapse
15	68	M	M4	46, XY	asp835	wt	MF
16	53	M	M5	nt	wt	Ins TCTG(A)	*De novo*
17	79	M	M5	nt	wt	wt	*De novo*
18	75	F	M4	46,XX	ITD	wt	*De novo*
19	45	F	M4	46,XX	wt	Ins TAGG	*De novo*
20	75	F	M1	nt	ITD	wt	*De novo*
21	82	M	M0	nt	wt	wt	PV
22	63	F	M4	46,XX	ITD	wt	*De novo*
23	76	M	M0	46,XY	wt	wt	*De novo*
24	46	M	M4	46,XY	ITD	Ins TGCA	Relapse
25	32	F	M5	del(5q)	wt	Ins TCTG(A)	*De novo*
26	29	M	M4	46,XY	ITD	Ins TCTG(A)	Relapse
27	62	M	M4	+8	wt	nt	*De novo*
28	68	M	M2	Complex	wt	wt	*De novo*
29	59	F	M2	-7	wt	wt	*De novo*
30	82	M	M2	nt	wt	wt	*De novo*
31	49	F	M2	Complex	wt	wt	CML + relapse
32	82	M	M5	- Y	wt	wt	*De novo*
33	81	F	M1	46,XX	wt	wt	MDS
34	33	M	M1	46,XY	wt	wt	*De novo*
35	35	M	M2	46,XY	wt	wt	*De novo*
36	48	M	M4	inv(16)	wt	wt	*De novo*
37	59	F	M4	46,XX	ITD	Ins TCTG(A)	Relapse
38	36	M	M5	inv(16), +8, +22	ITD	wt	*De novo*
39	70	F	M4	46,XX	wt	Ins TGCA	*De novo*
40	60	M	M4	46,XY	ITD	wt	*De novo*
41	84	M	M1	Complex	wt	wt	*De novo*
42	59	F	M4	46,XX	ITD	Ins	*De novo*
43	72	F	M1	nt	nt	nt	*De novo*

FAB = French-American-British classification; FLT3 = fms-related tyrosine kinas 3; wt = wild type; mut = mutant; Ins = insertion; MDS = myelodysplastic syndrome; nt = not determined; PV = polycythemia vera; CML = chronic myeloid leukemia.

Supplementary Table ~~3~~2. IC50 values of primary AML patient samples

Patient No.	FAB	Cytogenetics	FLT3	NPM1	Previous disease	IC50, µM
1	M2	Complex	wt	wt	CML	0.5
2	M5	46,XY	wt	wt	*De novo*	3.6
3	M1	nt	ITD	wt	*De novo*	0.7
5	M1	46,XX	nt	nt	*De novo*	27.3
7	M5	Complex	wt	Ins	*De novo*	19.4
8	M2	-7	wt	Ins	*De novo*	110.3 (res)
10	M5	46,XX	ITD	Ins	*De novo*	6.4
11	M1	Complex	wt	wt	MDS	0.6
13	M5	t(9:11),+19	wt	wt	*De novo*	1.1
18	M4	46,XX	ITD	wt	*De novo*	27.9
19	M4	46,XX	wt	Ins	*De novo*	7.5
24	M4	46,XY	ITD	Ins	Relapse	1.4
25	M5	del(5q)	wt	Ins	*De novo*	788 (res)
26	M4	46,XY	ITD	Ins	Relapse	207 (res)
27	M4	+8	wt	nt	*De novo*	0.8
28	M2	Complex	wt	wt	*De novo*	5.8
30	M2	nt	wt	wt	*De novo*	53.7
31	M2	Complex	wt	wt	CML	3.1
32	M5	-Y	wt	wt	*De novo*	17.1
33	M1	46,XX	wt	wt	MDS	11.1
36	M4	inv(16)	wt	wt	*De novo*	1.8
41	M1	Complex	wt	wt	*De novo*	0.5
42	M4	46,XX	ITD	Ins	*De novo*	0.3
43	M1	nt	nt	nt	*De novo*	71340(res)

FAB = French-American-British classification; FLT3 = fms-related tyrosine kinas 3; wt = wild type; mut = mutant; Ins = insertion; MDS = myelodysplastic syndrome; nt = not determined; CML = chronic myeloid leukemia; res = resistant.

Supplementary Table 43. Characteristics of AML patient samples; 7 days assay with cytokines.

Patient no.	Age	Sex	FAB	Cytogenetics	FLT3	NPM-1 (type mutation)	Previous disease
1	24	M	M2	Complex	wt	wt	CML
2	58	M	M5	46,XY	wt	wt	*De novo*
3	77	F	M1	nt	ITD	wt	*De novo*
6	65	M	M5	Complex	wt	Ins TCTG(A)	*De novo*
**7**	29	F	M5	46,XX	ITD,Asp835	wt	*De novo*
10	60	F	M5	46,XX	ITD	Ins TCTG(A)	*De novo*
13	67	F	M5	t(9:11),+19	wt	wt	*De novo*
**17**	79	M	M5	nt	wt	wt	*De novo*
18	75	F	M4	46,XX	ITD	wt	*De novo*
19	45	F	M4	46,XX	wt	Ins TAGG	*De novo*
20	75	F	M1	nt	ITD	wt	*De novo*
23	76	M	M0	46,XY	wt	wt	*De novo*
25	32	F	M5	del(5q)	wt	Ins	*De novo*
29	59	F	M2	-7	wt	wt	*De novo*
30	82	M	M2	nt	wt	wt	*De novo*
31	49	F	M2	Complex	wt	wt	CML+relapse
33	81	F	M1	46,XX	wt	nt	MDS
34	33	M	M1	46,XY	wt	wt	*De novo*
35	35	M	M2	46,XY	wt	wt	*De novo*
36	48	M	M4	inv(16)	wt	wt	*De novo*
37	59	F	M4	46,XX	ITD	Ins	*De novo*
38	36	M	M5	inv(16), +8, +22	ITD	wt	*De novo*
39	70	F	M4	46,XX	wt	Ins TGCA	*De novo*
39	70	F	M4	nt	wt	Ins TCTG(A)	MDS
40	60	M	M4	46,XY	ITD	wt	*De novo*
41	84	M	M1	Complex	wt	wt	*De novo*
42	59	F	M4	46,XX	ITD	Ins	*De novo*
43	72	F	M1	nt	nt	nt	*De novo*
44	67	M	M0	del(5q)	ITD	wt	*De novo*
45	71	M	M2	46,XY	asp835	Ins TCTG(A)/CCTG(D)	Relapse
46	72	M	M5	46,XY	wt	Ins TCTG(A)	*De novo*
47	43	M	M1	46,XY	wt	wt	*De novo*
48	64	F	M2	46,XX	ITD	Ins	Relapse
49	72	M	M4	46,XY	ITD	wt	*De novo*
50	36	M	M5	Complex	ITD	wt	*De novo*
51	81	F	M2	nt	ITD	Ins TCTG(A)	*De novo*
52	42	F	M5	46,XX	wt	Ins TCTG(A)	*De novo*
53	65	M	M4	46,XY	wt	wt	*De novo*
54	78	F	M0	Complex	wt	wt	*De novo*
55	80	F	M2	Complex	wt	wt	*De novo*
56	46	M	M1	46,XY	wt	Ins TCTG(A)	*De novo*
57	74	F	M2	nt	wt	wt	*De novo*

58	65	M	M5	46,XY	ITD	Ins TCTG(A)	*De novo*
59	68	M	M2	nt	ITD	Ins	MDS
60	82	F	M4	nt	ITD	wt	*De novo*
61	46	F	M1	inv(16)	wt	wt	*De novo*
62	56	F	M1	46,XX	wt	wt	*De novo*
63	64	M	M1	nt	ITD	Ins TCTG(A)	*De novo*
64	44	F	M1	del(7q)	ITD	wt	*De novo*
65	66	F	M1	46,XX	wt	Ins	*De novo*
66	67	M	M1	46,XY	ITD	wt	Relapse
67	61	M	M4	46,XY	ITD	Ins TCAG	*De novo*

FAB = French-American-British classification; FLT3 = fms-related tyrosine kinas 3; wt = wild type; mut = mutant; Ins = insertion; MDS = myelodysplastic syndrome; nt = not determined; CML = chronic myeloid leukemia.

Supplementary Table ~~5~~4. Characteristics of primary AML patient samples

Patient no.	Age		FAB	Cytogenetics	FLT3	NPM1 (Type mutation)	Previous disease
46	72	M	M5	46,XY	wt	Ins TCTG(A)	*De novo*
56	46	M	M1	46,XY	wt	Ins TCTG(A)	*De novo*
68	64	M	M5	46,XY	wt	Ins TCTG(A)	*De novo*
69	68	F	M5	46,XX	wt	Ins TCTG(A)	*De novo*

FAB = French-American-British classification; FLT3 = fms-related tyrosine kinase 3; wt = wild type; Ins = insertion.

Supplementary Table ~~6~~5. Concentration of BFA in plasma and tumor.

Time (h)	Plasma (nM)			Tumor (nM)		
	1	2	Mean	1	2	Mean
0	0,00	0,00	0,00	0,00	0,00	0,00
1	181,37	145,22	163,40	57,53	81,49	69,51
6	61.81	71,86	66,94	30,58	42,77	37,21
24	13,92	9,52	11,76	16,68	21,11	18,80

Snap-PK for 2 mg/kg BFA (Figure 7A) in two BALB/c mice (1,2), BFA: M.W. 467.56.
